# KLRG1 and NKp46 discriminate subpopulations of human CD117^+^CRTH2^−^ ILCs biased toward ILC2 or ILC3

**DOI:** 10.1084/jem.20190490

**Published:** 2019-06-14

**Authors:** Maho Nagasawa, Balthasar A. Heesters, Chantal M.A. Kradolfer, Lisette Krabbendam, Itziar Martinez-Gonzalez, Marjolein J.W. de Bruijn, Korneliusz Golebski, Rudi W. Hendriks, Ralph Stadhouders, Hergen Spits, Suzanne M. Bal

**Affiliations:** 1 Department of Experimental Immunology, Amsterdam UMC, Location AMC, University of Amsterdam, Amsterdam, The Netherlands; 2 Amsterdam Infection & Immunity Institute, Amsterdam, The Netherlands; 3 Department of Pulmonary Medicine, Erasmus MC, Rotterdam, The Netherlands; 4 Department of Cell Biology, Erasmus MC, Rotterdam, The Netherlands

## Abstract

Recently, human ILCs that express CD117 and CD127 but lack CRTH2 and NKp44 have been shown to contain precursors of ILC1, ILC2, and ILC3. However, these ILCs have not been extensively characterized. We performed an unbiased hierarchical stochastic neighbor embedding (HSNE) analysis of the phenotype of peripheral blood CD117^+^ ILCs, which revealed the presence of three major subsets: the first expressed NKp46, the second expressed both NKp46 and CD56, and the third expressed KLRG1, but not NKp46 or CD56. Analysis of their cytokine production profiles and transcriptome revealed that NKp46^+^ ILCs predominantly develop into ILC3s; some of them can differentiate into ILC1/NK-like cells, but they are unable to develop into ILC2s. In contrast, KLRG1^+^ ILCs predominantly differentiate into ILC2s. Single-cell cultures demonstrate that KLRG1^+^ ILCs can also differentiate into other ILC subsets depending on the signals they receive. Epigenetic profiling of KLRG1^+^ ILCs is consistent with the broad differentiation potential of these cells.

## Abstract (precis)

The human CD117^+^ CRTH2^−^ ILC population contains ILC precursors. Nagasawa et al. segregate this population by the mutually exclusive expression of KLRG1 and NKp46. KLRG1^+^ ILCs are biased toward the ILC2 lineage, whereas NKp46 clearly defines ILC3-lineage–biased cells.

## Introduction

Innate lymphoid cells (ILCs) exert their effector functions most prominently in tissues, particularly at mucosal sites. ILCs are rapidly activated by various stimuli produced by other immune and nonimmune cells, and this allows for an efficient response to the acute phase of infections and tissue damage (Artis and Spits, 2015; Ebbo et al., 2017). Consequently, ILCs are considered important in the maintenance and surveillance of mucosal integrity. ILCs have been categorized into five subsets based on their developmental trajectory, transcription factor (TF) requirements, and cytokine production profiles (Vivier et al., 2018). These are natural killer (NK) cells, ILC1s, ILC2s, ILC3s, and lymphoid tissue inducer cells. The ILC1, ILC2, and ILC3 subsets derive from a common precursor and express CD127 (IL-7Rα; Scoville et al., 2016); ILC1s are CD117^−^ cells that produce IFN-γ and depend on the TF T-bet; ILC2s express CRTH2, are capable of producing IL-5 and IL-13, and depend on GATA3; and ILC3s are CD117^+^ cells that can express natural cytotoxicity receptors, secrete IL-17 and IL-22, and require RAR-related orphan receptor (ROR)γt.

In addition to mucosal surfaces, ILCs can also be found in peripheral blood (PB). PB from healthy individuals consists of CRTH2^+^ ILC2s, CD117^−^CRTH2^−^ ILCs, and CD117^+^CRTH2^−^ ILCs. The CD117^+^CRTH2^−^ population was recently shown to consist of uni- and multipotent precursors of mature ILC1, ILC2, ILC3, and NK-like cells (Lim et al., 2017). Consistent with their differentiation potential, CD117^+^CRTH2^−^ ILCs express high levels of TFs that are essential for ILC development, such as inhibitor of DNA binding protein 2 (*ID2*), *GATA3*, thymocyte selection–associated high mobility group box protein (*TOX*), and TF7 (*TCF7*). In contrast, moderate to low levels of the lineage-determining TFs *RORC*, T-BOX 21 (*TBX21*), Eomesodermin (*EOMES*), cytokine receptors, and signature cytokines were found. A recent study provided additional information on ILC subsets in general by broad analysis of ILC surface antigen expression across several tissues and PB (Simoni et al., 2017). This study confirmed and extended earlier analyses of our group (Hazenberg and Spits, 2014; Mjösberg and Spits, 2016) that ILC2s and ILC3s can be characterized by high expression of CD127 and CD161; additional expression of CRTH2 and killer cell lectin-like receptor subfamily G member 1 (KLRG1) identified ILC2s, whereas natural cytotoxicity receptors NKp44, NKp46, and CD56 discriminate ILC3s (Simoni et al., 2017). A detailed analysis of PB ILCs based on the expression profiles reported in these studies may provide additional information on ILC heterogeneity and unravel their developmental process.

In the present study, we performed a flow cytometric analysis with an extensive panel of cell-surface markers and observed that the CD117^+^CRTH2^−^NKp44^−^ ILC population can clearly be segregated by the expression of KLRG1 and NKp46. We used a combination of gene expression profiling, epigenetic analyses, and differentiation assays to address the developmental status of these ILC subpopulations. These analyses showed that KLRG1-expressing ILCs represent a developmentally transitional stage of ILC2s, whereas the NKp46-expressing ILCs relate to IL-22–producing ILC3s. Furthermore, a subgroup of NKp46^+^ ILCs are capable of differentiation toward ILC1/NK-like cells that produce IFN-γ.

## Results

### Hierarchical stochastic neighbor embedding (HSNE) analysis of PB ILCs

**Figure 1.**
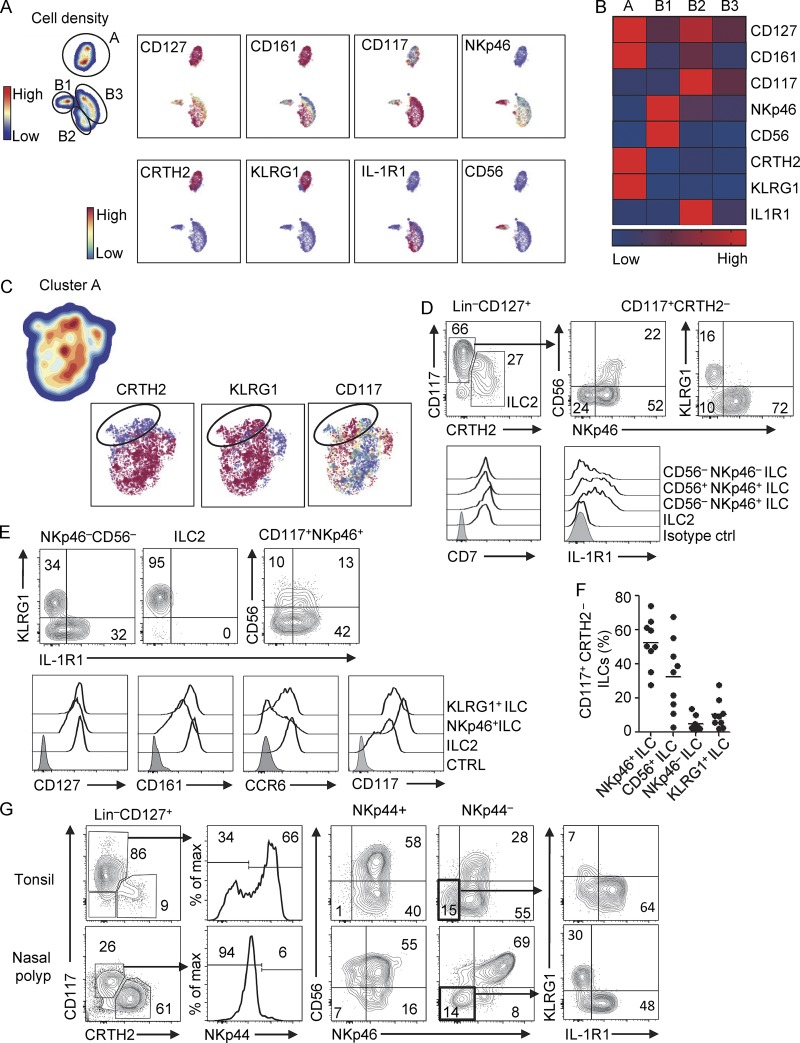
**Characterization of PB ILCs. (A)** HSNE analysis of the ILC population in PB (*n* = 8). Total PB lymphocytes were stained with antibodies against Lin (CD1a, CD3, CD4, CD5, CD14, CD19, CD16, CD34, CD94, CD123, BDCA2, TCRαβ, TCRγδ, and FcER1α) and ILC-related molecules as indicated. The Lin^−^CD127^+^ population (ILCs) was further analyzed to identify clusters based on the expression of different cell-surface molecules. Two clusters are indicated as A and B, and cluster B is subdivided into three subclusters (B1, B2, and B3). **(B)** Heatmap of expression intensity of cell-surface molecules on different ILC clusters. **(C)** Zoom-in of cluster A by HSNE. The circle indicates a population that expresses KLRG1 but lacks CRTH2. **(D)** Gating strategy for flow cytometric analysis of PB ILC subsets (three upper plots) and histogram of CD7 and IL1R1 expression on ILC subsets (bottom). **(E)** KLRG1, CD56, and IL1R1 expression pattern on ILC subsets (three upper plots), and histogram of several ILC associated cell-surface molecules on KLRG1^+^ ILCs (Lin^−^CD127^+^CD117^+^CRTH2^−^NKp46^−^KLRG1^+^), ILC2s (Lin^−^CD127^+^CRTH2^+^), and NKp46^+^ ILCs (Lin^−^CD127^+^CD117^+^CRTH2^−^CD56^−^NKp46^+^; bottom). Filled histogram represents isotype control (CTRL). **(F)** Frequency of each subset indicated within the CD117^+^ CRTH2^−^ ILC population from PB (*n* = 9). **(G)** Gating strategy used for flow cytometric analysis of ILC subsets in NPs and tonsils. Data in D, E, and G are representative of at least three donors from at least three independent experiments.

Recent studies have shown that the CD117^+^ PB ILCs contain multi- and unipotent ILC precursors (Lim et al., 2017; Chen et al., 2018). Those observations prompted us to perform an extensive flow cytometric analysis of CD117^+^CRTH2^−^ ILCs in an attempt to define subsets within this population. We analyzed blood lymphocytes from eight healthy donors by 13-color flow cytometry. Single-cell suspensions were stained with a lineage (Lin) antibody cocktail (see Materials and methods) and multiple antibodies against ILC-associated molecules. The Lin^−^CD94^−^CD127^+^ total ILC population was then analyzed by HSNE (van Unen et al., 2017). This unbiased approach identified two major clusters (A and B), of which cluster B could be subdivided into three further subclusters (B1, B2, and B3), also in an unbiased matter. The subclusters could be defined by the expression of NKp46, CD56, and IL1R1, all ILC3-associated proteins (Cupedo et al., 2009; Cella et al., 2010; Hughes et al., 2010; Scoville et al., 2016; Fig. 1, A–C; and Fig. S1 A). Cells within cluster A uniformly expressed higher levels of CD127 and CD161; additionally, they exclusively expressed CRTH2 and/or KLRG1 (Fig. 1, A and B). Based on this analysis, we conclude that cluster A represents PB ILC2s and cluster B contains ILC3-related cells. Interestingly, although CRTH2 is reported to be the ILC2-defining marker, the HSNE analysis suggested the presence of a CRTH2^−^ ILC2 population that expresses KLRG1 and has similar CD117 expression as CRTH2^+^ ILC2s (Fig. 1 C). Subcluster B1 differed from B2 and B3 by lower CD117 and higher CD56 and NKp46 expression. However, cells in subcluster B1 were clearly distinct from CD56^+^CD127^−^ NK cells, as they have higher expression of CD117 and CD200R, similar to CD56^−^CD127^+^ ILCs (Fig. S1 B). Previously, it was demonstrated that CD200R distinguishes mouse ILC1s from NK cells (Weizman et al., 2017). Here, we demonstrate that CD200R is expressed not only on ILC1s but also on all PB ILCs, indicating that this marker discriminates all human ILC subsets from NK cells. The CD56^+^CD200R^+^ ILCs are probably similar to the CD56^+^ ILCs that were recently described in tonsils to give rise to NK cells, ILC1s, and ILC3s (Chen et al., 2018). HSNE analysis of total CD127^+^ ILCs in tonsil confirmed the presence of all PB ILC subsets as well as NKp44^+^ ILC3s (Fig. S1 C).

### CRTH2^−^CD117^+^ ILCs enclose KLRG1^+^ and NKp46^+^ populations

The four distinct ILC populations in PB, identified by HSNE analysis, were resolved by classical flow cytometry to better visualize low-frequency cell populations. Cluster A clearly contained KLRG1-expressing cells that lack CRTH2 (Fig. 1 C). After segregating the major ILC subsets by CD117 and CRTH2, CD117^+^CRTH2^−^ ILCs were further analyzed for expression of NKp46, KLRG1, and CD56, which separated them into four populations (Fig. 1 D). The four ILC populations were identified as: KLRG1^+^NKp46^−^CD56^−^ (KLRG1^+^ ILCs), KLRG1^−^NKp46^−^CD56^−^ (NKp46^−^ ILCs), KLRG1^−^NKp46^+^CD56^−^ (NKp46^+^ ILCs), and KLRG1^−^NKp46^+^CD56^+^ (CD56^+^ ILCs), which uniformly express CD7 in line with previously reported phenotypes (Fig. 1 D; Lim et al., 2017). KLRG1^+^ ILCs did not express IL1R1, whereas NKp46^+^ ILCs and some NKp46^−^ ILCs expressed this receptor, verifying the HSNE analysis (Fig. 1, D and E). KLRG1^+^ ILCs and ILC2s showed similar expression of CD127, CD161, and CCR6, supporting the notion that KLRG1^+^ ILCs are more related to ILC2s than to ILC3s (Fig. 1 E). Analysis of ILC subset frequency showed that NKp46^+^ ILCs were most prevalent (52% of all PB CD117^+^CRTH2^−^ ILCs), followed by CD56^+^ ILCs (33%), NKp46^−^ ILCs (9%), and KLRG1^+^ ILCs (5%). All populations could be detected in all donors (Fig. 1 F).

These CD117^+^CRTH2^−^ ILC populations were not only present in PB but also in inflamed tonsils and nasal polyp (NP) tissue from patients with chronic rhinosinusitis (CRS; Figs. 1 G and S1 D). ILC3s are enriched in tonsils, while ILC2s are dominant in NP (Mjösberg et al., 2011). Unlike PB, these tissues contain NKp44-expressing ILC3s, which coexpress NKp46 and partly CD56 (Figs. 1 G and S1 D). KLRG1^+^ ILCs were present exclusively in the NKp44^−^NKp46^−^ population, although the frequency was clearly higher in NPs as compared with tonsils, which may reflect the frequency of ILC2 in these tissues (Figs. 1 G and S1 E).

### The expression pattern of CRTH2, KLRG1, NKp46, and NKp44 defines different developmental stages of ILCs

**Figure 2.**
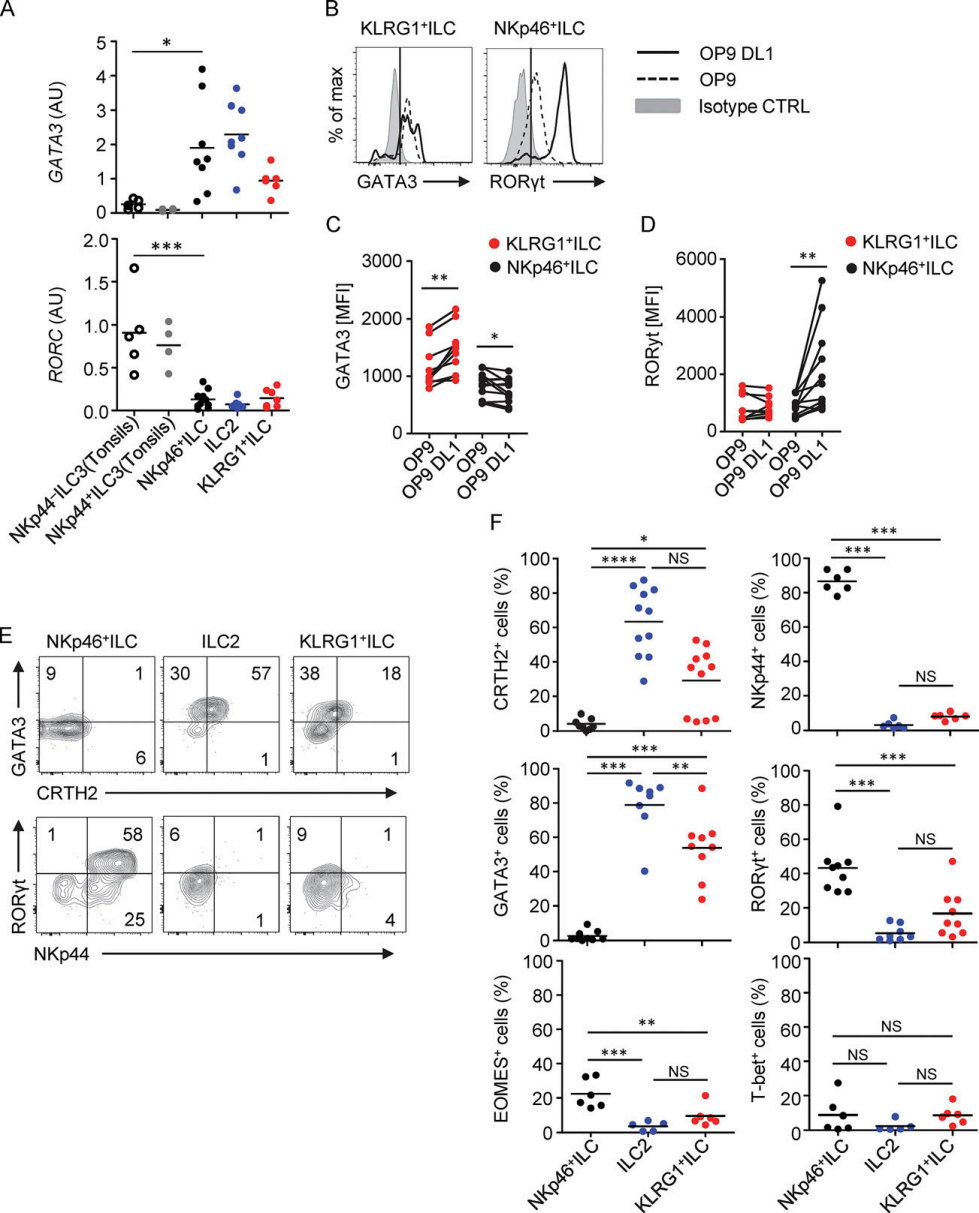
**Differentiation capacity of KLRG1^+^ ILCs and NKp46^+^ ILCs. (A)** Quantification of relative *GATA3* and *RORC* expression as compared with *GAPDH* in different ILC subsets isolated from tonsils and PB. **(B)** Representative histograms of GATA3 and RORγt expression in KLRG1^+^ ILCs and NKp46^+^ ILCs, respectively, after culture for 5 d on OP9 or OP9-DL1 in the presence of IL-2 (20 U/ml) and IL-7 (20 ng/ml). **(C)** Quantification of GATA3 expression on KLRG1^+^ and NKp46^+^ ILCs after culture as in B. **(D)** Quantification of RORγt expression on KLRG1^+^ and NKp46^+^ ILCs after culture as in B. **(E)** Representative flow cytometric analysis of CRTH2, GATA3, NKp44, and RORγt expression on KLRG1^+^ ILCs, ILC2s, and NKp46^+^ ILCs after culture for 5 d on OP9-DL1 in the presence of IL-2 (20 U/ml) and IL-7 (20 ng/ml). **(F)** Quantification of the percentage of cells expressing CRTH2, NKp44, GATA3, RORγt, EOMES, and T-bet upon culture as in E. Each dot represents one individual donor. *, P < 0.05; **, P < 0.001; ***, P < 0.0001; ****, P < 0.00001 (one-way ANOVA). Data in B and E are representative of at least three donors from at least three independent experiments. AU, arbitrary units; CTRL, control; MFI, mean fluorescence intensity.

To characterize the KLRG1^+^ ILCs and NKp46^+^ ILCs in more detail, we evaluated the expression of ILC2 and ILC3 signature TFs. As expected, all PB ILCs expressed *GATA3*. In addition, all PB ILCs expressed low levels of *RORC* (Fig. 2 A). Both NKp44^−^NKp46^+^ ILC3s and NKp44^+^NKp46^+^ ILC3s from tonsil expressed very low levels of *GATA3* but high levels of *RORC* (Fig. 2 A). To assess the differentiation capacity of KLRG1^+^ ILCs and NKp46^+^ ILCs, we used a stromal cell–based culture system that supports ILC expansion and differentiation (Lim et al., 2016, 2017; Scoville et al., 2016). Isolated KLRG1^+^ ILCs and NKp46^+^ ILCs were cultured on OP9 cells, which either did or did not express the Notch ligand delta-like (DL)1. To evaluate their unbiased developmental capacity, only IL-2 and IL-7 were added without additional inflammatory cytokines. After 5 d of culture, GATA3 was detected in KLRG1^+^ ILCs regardless of the presence of a Notch ligand, whereas RORγt was strongly up-regulated in NKp46^+^ ILCs in a Notch-signaling–dependent manner (Fig. 2, B–D). Further cultures were performed on OP9-DL1 cells, as these cells promoted the differentiation of both ILC2s and ILC3s. In this setting, we observed that besides the lineage-determining TFs, KLRG1^+^ ILCs from PB and NP were able to up-regulate CRTH2, whereas NKp46^+^ ILCs did not (Fig. 2, E and F; and Fig. S2 A). CRTH2 expression was not consistently up-regulated on KLRG1^+^ ILCs in each donor. The reason for the heterogeneity is unclear, but we noted that in those donors from whom the KLRG1^+^ ILCs failed to up-regulate CRTH2, this receptor was down-regulated when CRTH2^+^ ILC2s were cultured under the same conditions (Fig. 2 F). We also observed that the expression level of CRTH2, induced on KLRG1^+^ ILCs, cultured with IL-2 and IL-7 was lower than that of ILC2s (Fig. 2 F), suggesting that the experimental conditions may not be optimal to support full CRTH2 induction.

NKp46^+^ ILCs from PB and NKp44^−^ ILC3 from tonsil differentiated into NKp44^+^ RORγt^+^ ILC3s (Fig. 2, E and F; and Fig. S2 B). KLRG1^+^ ILCs could also differentiate in some cases to RORγt^+^ ILCs, but these cells failed to up-regulate NKp44. In line with previous observations showing that CD117^+^CRTH2^−^ PB ILCs can differentiate into NK-like cells (Lim et al., 2017), NKp46^+^ ILCs cultured under this condition maintained NKp46 expression and up-regulated CD56, and some of them gave rise to EOMES-expressing cells (Fig. 2 F and Fig. S2 C). These EOMES^+^ cells coexpressed CD94, but not RORγt, T-bet, or CD16 (Fig. S2, C and D). We observed that tonsil NKp44^−^ ILC3s, but not NKp44^+^ ILC3s, could also give rise to EOMES^+^ RORγt^−^ cells (Fig. S2 B). These in vitro–differentiated CD56^+^ cells are phenotypically similar to ex vivo PB NKp46^+^CD56^+^ ILCs, as they express *EOMES* and very low *TBX21* (T-bet). As NKp46^+^CD56^+^ cells ex vivo lack expression of Granzyme B (*GZMB*) and Perforin 1 (*PRF1*) and express significantly higher levels of *RORC* than NK cells (Fig. S2 E), EOMES^+^NKp46^+^CD56^+^ cells are clearly different from conventional NK cells.

### KLRG1^+^ and NKp46^+^ ILCs are biased to differentiate into mature ILC2s and ILC3s, respectively

**Figure 3.**
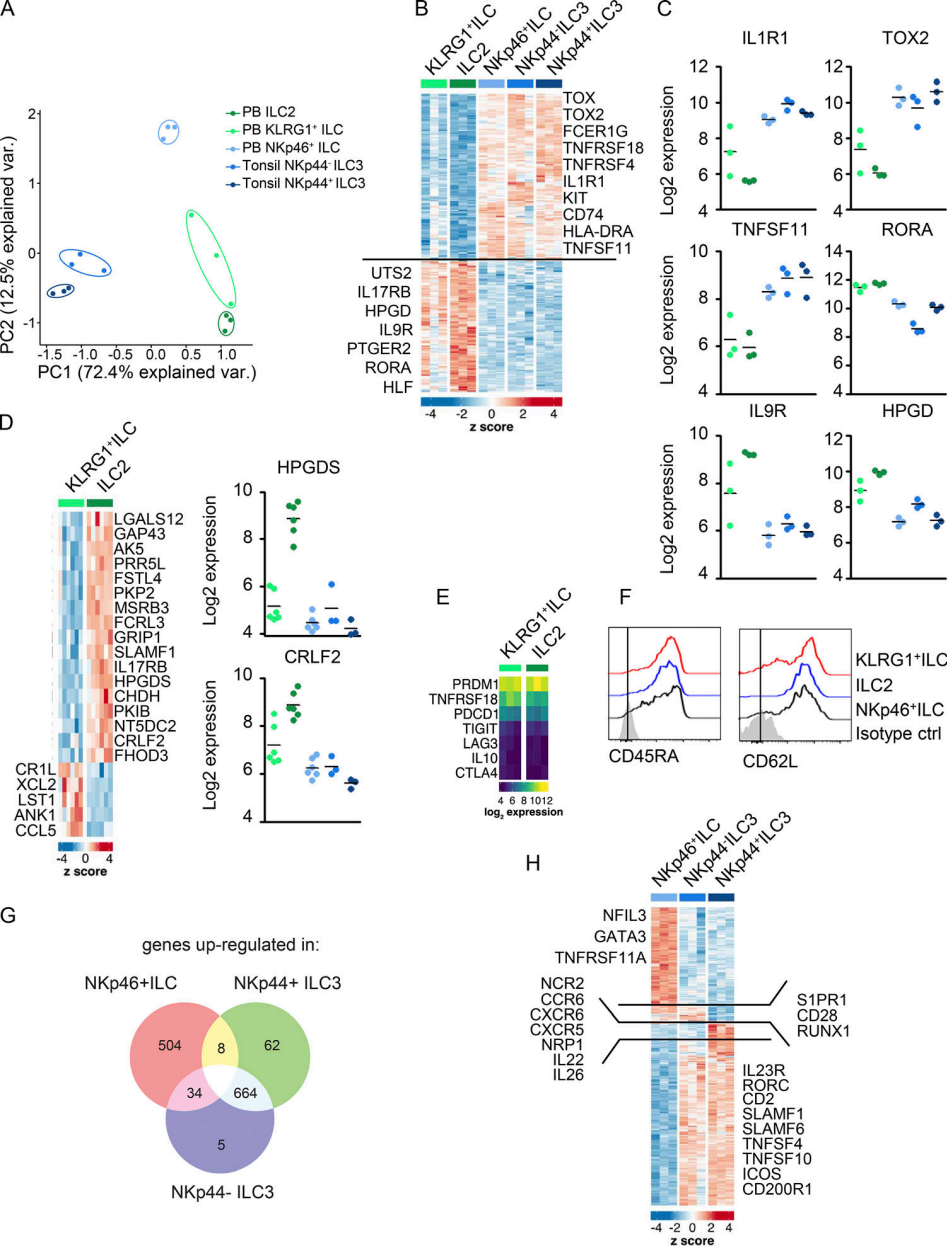
**Microarray analysis of KLRG1^+^ ILCs, NKp46^+^ ILCs, ILC2s, tonsil NKp44^−^ ILC3s, and NKp44^+^ ILC3s. (A)** PCA plot of gene expression as determined by microarray in KLRG1^+^ ILCs, ILC2s, NKp46^+^ ILCs, tonsil isolated NKp44^−^ ILC3s, and NKp44^+^ ILC3s; three donors each). **(B)** Heatmap of significantly different expressed genes associated with ILC2s and ILC3s. **(C)** Log_2_ expression showing the expression values of selected genes. **(D)** Heatmap of significantly differently expressed genes between KLRG1^+^ ILCs and ILC2s (left) and log_2_ expression of HPGDS and CRLF2 (right). **(E)** Heatmap of expression of genes related to ILC2 exhaustion. **(F)** Representative histograms of expression of CD62L and CD45RA on KLRG1^+^ ILCs, NKp46^+^ ILCs, and ILC2s. **(G)** Venn diagram showing genes that are up-regulated in NKp46^+^ ILCs, tonsil NKp44^−^ ILC3s, and tonsil NKp44^+^ ILC3s. **(H)** Heatmap of significantly different expressed genes between NKp46^+^ ILCs, tonsil NKp44^−^ ILC3s, and tonsil NKp44^+^ ILC3s. Data in F are representative of at least three donors from at least three independent experiments. PC, principal component; HLA-DRA, human leukocyte antigen DR alpha; HLF, hepatic leukemia factor; HPGD, 15-hydroxyprostaglandin dehydrogenase; HPGDS, hematopoietic prostaglandin D synthase; KIT, proto-oncogene, receptor tyrosine kinase; RORA, RAR-related orphan receptor.

Next, we characterized the transcriptomes of the KLRG1^+^ ILC and NKp46^+^ ILC subsets by microarray analysis. Principal-component analysis (PCA) based on the most variable genes (interquartile range [IQR] >1.5) showed a clear separation of KLRG1^+^ ILCs, NKp46^+^ ILCs, ILC2s, and tonsil NKp44^−^ ILC3s and NKp44^+^ ILC3s, although one donor of KLRG1^+^ ILCs clustered more with ILC2s (Fig. 3 A and Table S1 A). Analysis of the most differently expressed genes revealed that KLRG1^+^ ILCs and ILC2s shared 108 genes that were expressed higher as compared with NKp46^+^ ILCs, NKp44^−^ ILC3s, and NKp44^+^ ILC3s, including RORα, one of the TFs known to be essential for ILC2 development in mice (Wong et al., 2012; Fig. 3, B and C; and Table S1 B). KLRG1^+^ ILCs and ILC2s also shared higher expression levels of *IL9R* (IL-9 receptor), *IL17RB* (IL-25 receptor), *PTGER2* (prostaglandin E2 receptor), and *HPGD* (15-hydroxyprostaglandin dehydrogenase), all markers that are typically associated with the ILC2 lineage (Fig. 3, B and C; Turner et al., 2013; Björklund et al., 2016). The similarity of ILC2s and KLRG1^+^ ILCs was further emphasized by the observation that only 22 genes were significantly differently expressed (Fig. 3 D and Table S1 C). KLRG1^+^ ILCs had significantly lower expression of *CRLF2* (TSLP receptor), *FCRL3*, and *HPGDS*, all genes that have been previously associated with ILC2s (Björklund et al., 2016; Gury-BenAri et al., 2016). This could be a consequence of the lower *GATA3* expression in KLRG1^+^ ILCs (Fig. 2 A), since these genes were suggested to be targets of GATA3 (Björklund et al., 2016). KLRG1^+^ ILCs had significantly higher expression of two chemokine genes, *XCL2* and *CCL5* (RANTES); *ANK1* (Ankyrin-1, a membrane protein), which plays a role in cell stability and mobility; *CR1L* (complement component receptor 1-like protein); and *LST1* (lymphocyte-specific transcripts 1), which can inhibit the proliferation of lymphocytes (Fig. 3 D). Expression of IL-5 and IL-13, ILC2 signature cytokines, was not detected in KLRG1^+^ ILCs nor in ILC2s. This indicates that these cells are in a resting state and produce these cytokines only after activation, as has also been demonstrated for tonsil ILC2s (Björklund et al., 2016). It is unlikely that the KLRG1^+^ ILCs are either exhausted or ex-ILC2s, as the expression of ILC2 exhaustion markers, as described by Miyamoto et al. (2019), does not differ between ILC2s and KLRG1^+^ ILCs (Fig. 3 E). Additionally, both express CD62L and CD45RA, which are expressed on resting ILCs (Fig. 3 F; Bar-Ephraim et al., 2019).

We found 138 genes, shared by NKp46^+^ ILCs and NKp44^−^ and NKp44^+^ ILC3s from tonsils, that were significantly higher expressed as compared with ILC2s and KLRG1^+^ ILCs (Fig. 3 B). These included ILC3-associated TFs, such as *TOX*, *TOX2*, and *RUNX2*, and the cell-surface markers *IL1R1*, *TNFRSF18* (GITR), *TNFRSF4* (OX40), and *TNFSF11* (receptor activator of nuclear factor kappa-Β ligand [RANKL]; Fig. 3, B and C; and Table S1 D; Björklund et al., 2016; Gury-BenAri et al., 2016; Bar-Ephraim et al., 2017). NKp46^+^ ILCs and the two tonsillar ILC3 populations also shared the expression of genes associated with antigen presentation (*CD74*, *HLA-DRA*, *HLA-DPA1*, *HLA-DMB*, and *HLA-DMA*; Fig. 3 B and Table S1 D).

PB NKp46^+^ ILCs are more similar to tonsil ILC3s as compared with PB KLRG1^+^ ILCs. However, a more in-depth comparison of NKp46^+^ ILCs to NKp44^−^ ILC3s and NKp44^+^ ILC3s from tonsils revealed that they are still very distinct from tonsil NKp44^−^ ILC3s and NKp44^+^ ILC3s (34 and 8 shared genes, respectively). This is in stark contrast with NKp44^−^ ILC3s and NKp44^+^ ILC3s, which share expression of 664 genes (Fig. 3 G and Table S1 E). NKp46^+^ ILCs have higher expression of genes that are important for development of ILC3s such as *NFIL3* and *GATA3* as well as *TNFRSF11A* (RANK), a receptor regulating ILC3 suppression (Fig. 3 H and Table S1 F; Bando et al., 2018). NKp44^−^ and NKp44^+^ ILC3s from tonsils expressed higher levels of *RORC* and the prototypical ILC3 markers *IL23R*, *CD2*, *TNFSF4* (OX40L), and *TNFSF10* (TNF-related apoptosis-inducing ligand [TRAIL]), indicating that these cells are mature ILC3s. Only tonsillar NKp44^+^ ILC3s expressed high levels of *IL22, IL26*, and *TNFSF13B* (B-cell activating factor [BAFF]), suggesting that these cells were activated in situ (Bar-Ephraim et al., 2017). These NKp44^+^ ILC3s also expressed higher levels of the chemokine receptors *CCR6*, *CXCR6*, and *CXCR5* and the lymphoid tissue inducer cell marker *NRP1* (Shikhagaie et al., 2017). Interestingly, PB NKp46^+^ ILCs and NKp44^−^ ILC3s shared high expression of *S1PR1,* which is involved in ILC migration (Björklund et al., 2016; Bar-Ephraim et al., 2017). Altogether, these data show that KLRG1^+^ ILCs are more similar to ILC2s and NKp46^+^ ILCs to ILC3s, although genes associated with key functions of mature ILC3s, including signature cytokines, are not expressed in NKp46^+^ ILCs.

### KLRG1^+^ ILCs and NKp46^+^ ILCs can produce multiple cytokines when stimulated with inflammatory cytokines

**Figure 4.**
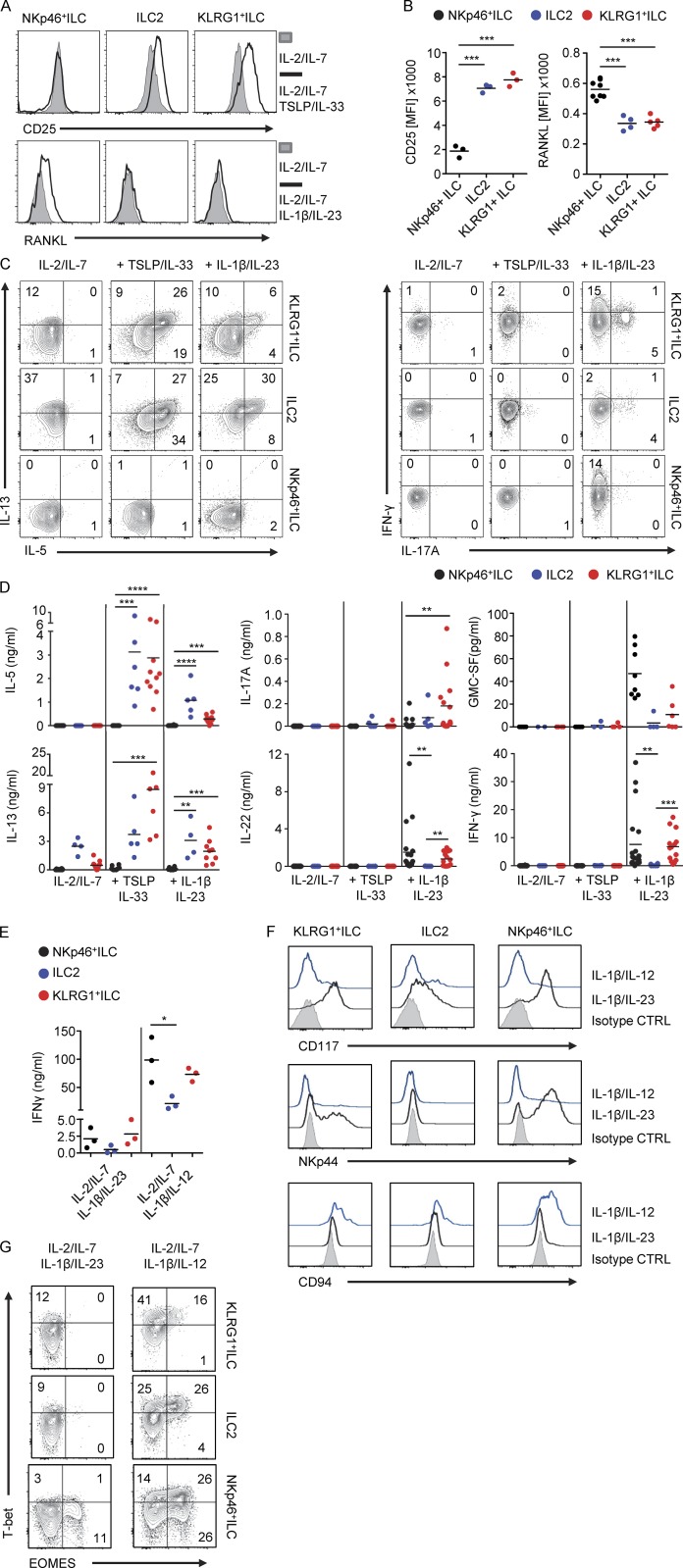
**KLRG1^+^ ILCs and NKp46^+^ ILCs show multipotent cytokine production and TF profile. (A)** Representative histogram of expression of CD25 or RANKL on KLRG1^+^ ILCs, ILC2s, and NKp46^+^ ILCs after culture on OP9-DL1 cells in the presence of IL-2 and IL-7 with or without TSLP and IL-33 or IL-1β and IL-23 for 7 d. **(B)** Quantification of CD25 or RANKL expression on after culture as in A (*n* = 3–8). **(C)** Representative flow cytometric analysis of intracellular IL-5, IL-13, IFN-γ, and IL-17A in KLRG1^+^ ILCs, ILC2s, and NKp46^+^ ILCs, after 7 d culture as in A and subsequently stimulated by PMA/ionomycin for 3 h. **(D)** Quantification of cytokine production by ELISA in culture supernatants from cells stimulated as in A. The concentration is adjusted to 5,000 cells. **(E)** Quantification of IFN-γ production by ELISA of ILC subsets cultured on OP9-DL1 cells in the presence of IL-2 and IL-7 with IL-1β and IL-23 or IL-1β and IL-12 for 7 d. **(F)** Representative flow cytometry of the expression of CD117, NKp44, and CD94 on KLRG1^+^ ILCs, ILC2, and NKp46^+^ ILCs after culture as in E. Filled histograms represent isotype control. **(G)** Representative flow cytometric analysis of intracellular expression of EOMES and T-bet in KLRG1^+^ ILCs, ILC2s, and NKp46^+^ ILCs after culture as in E. Data in A, C, F, and G are representative of at least three donors from more than three independent experiments. Cytokines used in all experiments are IL-2 (20 U/ml), IL-7, TSLP, IL-33, IL-1β, IL-23, and IL-12 (all 20 ng/ml). **, P < 0.001; ***, P < 0.0001; ****, P < 0.00001 (one-way ANOVA).

KLRG1^+^ ILCs and NKp46^+^ ILCs do not produce cytokines in the differentiation cultures with IL-2 and IL-7. To evaluate the functional capacities of KLRG1^+^ ILCs and NKp46^+^ ILCs, we co-cultured these ILCs and ILC2s from PB for 7 d on OP9-DL1 cells in the presence of different inflammatory cytokines. KLRG1^+^ ILCs and ILC2s up-regulated the ILC2-related activation molecule CD25 (Mjösberg et al., 2011) in response to TSLP and IL-33, whereas NKp46^+^ ILCs did not (Fig. 4, A and B). In contrast, the ILC3-associated molecule RANKL was specifically up-regulated only on NKp46^+^ ILCs in response to IL-1β and IL-23 (Fig. 4, A and B). In addition, both KLRG1^+^ ILCs and ILC2s produced IL-5 and IL-13 not only upon culture in the presence of TSLP and IL-33 but also in response to IL-1β and IL-23, although TSLP with IL-33 was a more potent inducer of these cytokines (Fig. 4, C and D). Interestingly KLRG1^+^ ILCs cultured on OP9-DL1 cells with IL-1β and IL-23 secreted not only IL-5 and IL-13 but also IFN-γ, IL-22, IL-17A, and GM-CSF (Fig. 4, C and D; and Fig. S2 F). Low amounts of IL-17A were also produced by ILC2s, but ILC2s produced no IFN-γ and IL-22 when stimulated with IL-1β and IL-23 (unlike KLRG1^+^ ILCs).

NKp46^+^ ILCs did not respond to IL-33 and TSLP or produce IL-5 and IL-13 but secreted IFN-γ, IL-22, and low amounts of IL-17A and GM-CSF upon IL-1β and IL-23 stimulation when cultured on OP9-DL1 (Fig. 4, C and D; and Fig. S2 F). The production of IFN-γ was increased upon culture of both NKp46^+^ and KLRG1^+^ ILCs with IL-1β and IL-12 (Fig. 4 E). This was accompanied by down-regulation of CD117 and NKp44 expression, indicating that these cells transdifferentiated into ILC1s in response to IL-12, as we have previously shown for CRTH2^+^ ILC2s and NKp44^+^ ILC3s (Fig. 4 F; Bernink et al., 2015; Bal et al., 2016). EOMES, T-bet, and CD94 were up-regulated in all PB ILC populations upon culture with IL-1β and IL-12, suggesting that this culture condition introduces NK cell features in all ILC subsets (Fig. 4, F and G).

### Clonal analysis of CD117^+^ PB ILC subsets shows broad functional differentiation capacities

**Figure 5.**
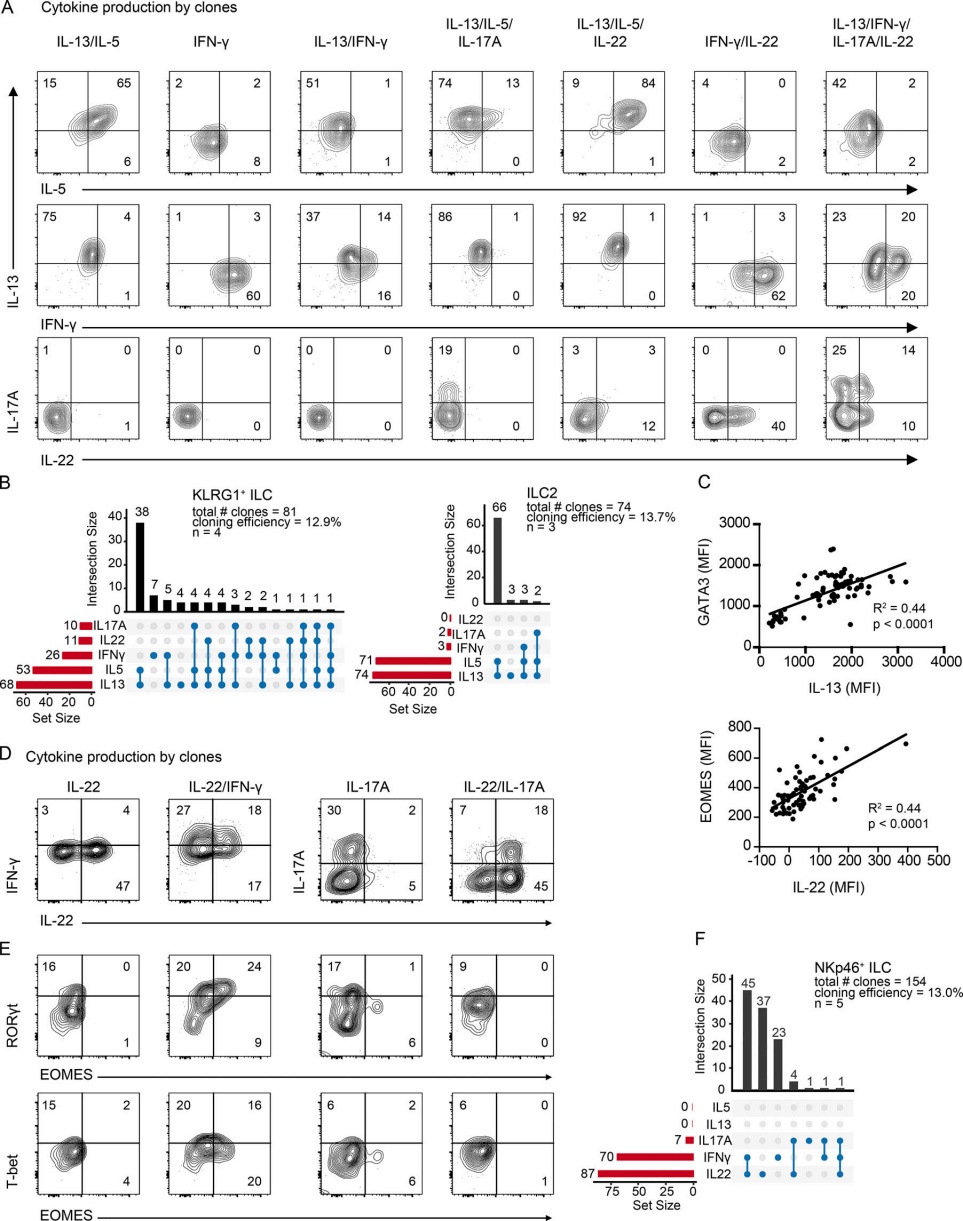
**Clonal analysis of CD117^+^ PB ILC subsets shows broad functional differentiation capacities. (A–E)** Single KLRG1^+^ ILCs, ILC2s, or NKp46^+^ ILCs from PB were index sorted by FACS into 96-well round-bottom plates preseeded with OP9-DL1 and stimulated with IL-2 (20 U/ml), IL-7, IL-1β, and IL-23 (20 ng/ml each). **(A)** After 14–21 d, KLRG1^+^ ILC cultures were analyzed for intracellular cytokine production (IL-5, IL-13, IFN-γ, IL-17A, and IL-22) after PMA/ionomycin stimulation. Representative flow cytometric analysis of clones producing various combinations of cytokines as indicated. **(B)** Summary of numbers and type of cytokines produced by KLRG1^+^ ILC clones and ILC2 clones. Three KLRG1^+^ ILC clones did not produce any of the measured cytokines. **(C)** Correlation of IL-13 production with GATA3 expression and IL-22 production with EOMES expression in KLRG1^+^ ILCs. **(D)** Representative flow cytometric analysis of NKp46^+^ ILC clones producing various combinations of cytokines as indicated. **(E)** Representative flow cytometric analysis of intracellular RORγt, T-bet, and EOMES expression of selected clones obtained from NKp46^+^ ILCs upon culture as in A. **(F)** Summary of numbers and type of cytokines produced by NKp46^+^ ILC clones. 28 NKp46^+^ ILC clones did not produce any of the measured cytokines. Data in A, C, and D are representative of at least four donors. MFI, mean fluorescence intensity.

The observation that KLRG1^+^ ILCs produced type 1, 2, and 3 cytokines following stimulation with IL-1β and IL-23 raised the possibility that IL-1β and IL-23 promote differentiation of KLRG1^+^ ILCs into multiple ILC subsets. Alternatively, the KLRG1^+^ ILCs may contain mixtures of clones with different precursor activities. To address this, we single-cell sorted KLRG1^+^ ILCs, NKp46^+^ ILCs, and ILC2s and cultured them on OP9-DL1 in the presence of IL-2, IL-7, IL-1β, and IL-23, as this culture condition promoted differentiation of all three PB ILC populations into cytokine-producing ILC subsets (Fig. 4, C and D). After 2 wk, with a cloning efficiency of ∼13%, all three subsets gave rise to cytokine-producing ILCs. From the 110 KLRG1^+^ ILC clones, 81 were evaluated for their cytokine production, surface protein, and TF expression. The cytokine production pattern differed among clones, and clones could be divided into single-, dual-, or multicytokine producers (Fig. 5 A). 68 clones (84%) produced IL-13; and 52 clones (64%) produced both IL-5 and IL-13, of which 38 (47%) did not coproduce other cytokines. These data indicate that most of the KLRG1^+^ ILC differentiated into IL-5– and IL-13–producing ILC2s. However, IL-17A–, IFN-γ–, and IL-22–producing clones were also detected, most of which coproduced IL-5 and/or IL-13, indicating that these cells retain ILC2 features but are more flexible (Fig. 5, A and B). A small minority of the KLRG1^+^ ILC-derived clones expressed only IFN-γ. IL-13 production correlated with GATA3 expression and IL-22 production with EOMES expression (Figs. 5 C and S3 A). ILC2s retained their ILC2 fate in response to IL-1β and IL-23, as 96% of ILC2s produced IL-13 and IL-5 and only 7% coproduced IL-17 or IFN-γ and no IL-22–producing clones were obtained (Fig. 5 B).

Analysis of the clones derived from NKp46^+^ ILCs revealed that the majority of clones produced IL-22 (82%) and a major part of IL-22 producing clones also coproduced IFN-γ (45%). Only a few clones (5%) produced IL-17A, confirming the results obtained in bulk cultures (Fig. 5, D and F). IFN-γ single-producing clones (15%) were also observed in this culture. No GATA3 was detected in any clones, similar to what we observed in the bulk culture, and RORγt was expressed in this culture condition (Fig. 5 E). Coexpression of RORγt, T-bet, and EOMES could be detected in clones coproducing IL-22 and IFN-γ, indicating that EOMES is not uniquely specific for human NK cells, at least under in vitro conditions. We also observed that IFN-γ single-producing clones are heterogeneous in terms of TF profiles, as they showed various combinations of RORγt, EOMES, and T-bet expression (Fig. S3 B). These results indicate that the majority of NKp46^+^ ILCs differentiated into IL-22–producing ILC3s, and some of them acquired IFN-γ production capacity. Not all IFN-γ–producing clones showed characteristics of ILC1/NK-like cells; instead, they displayed an intermediate ILC3- ILC1/NK-like phenotype. Collectively, our data suggest that KLRG1^+^ ILCs belong to the ILC2 lineage, whereas NKp46^+^ ILCs are skewed to ILC3s.

As the CD117^+^ ILC population also included a small population that did not express NKp46 or KLRG1, this population might contain a more upstream precursor that can give rise to both the KLRG1^+^ ILCs and NKp46^+^ ILCs. Indeed, bulk and clonal cultures revealed that this population was able to respond to IL-33/TSLP and IL-1β/IL-23. Clonal cultures with IL-1β and IL-23 resulted in production of IL-5, IL-13, IL-17A, IL-22, and IFN-γ (Fig. S3, C–E).

### Unique epigenetic features of KLRG1^+^ ILCs support their immature state

**Figure 6.**
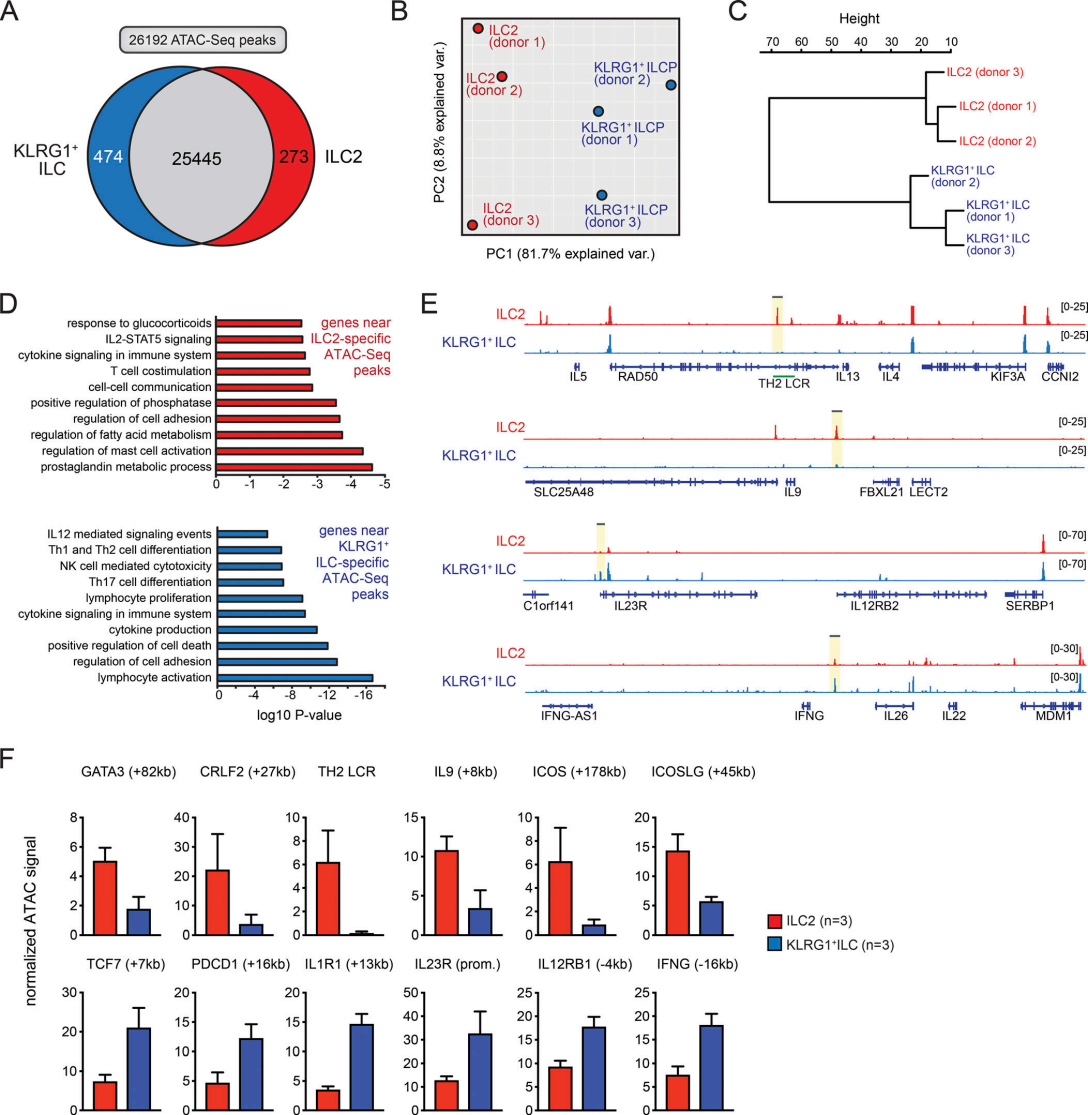
**Unique epigenetic features of KLRG1^+^ ILCs support their immature state**. **(A)** Venn diagram showing the total number of ATAC-seq peaks (*n* = 26,192, detected in at least two replicate datasets) and the subsets uniquely or substantially more accessible (>2.0-fold) in either ILC subset. **(B and C)** PCA (B) or hierarchical clustering (C) on the 747 differentially enriched ATAC-seq peaks defined in A. **(D)** Pathway enrichment analysis using the genes assigned to both sets of differentially enriched ATAC-seq peaks. **(E and F)** Representative genome browser shots of ATAC-seq signals across selected loci (E); signals in highlighted regions were quantified across the three biological replicate datasets generated (F). Error bars indicate SEM. PC, principal component; ICOS, inducible T cell costimulator; ICOSLG, inducible T cell costimulator ligand; IFNG, interferon gamma; LCR, locus control region.

The different gene expression and functional differentiation capabilities of KLRG1^+^ ILCs and ILC2s is likely reflected by differences in their epigenome, as epigenetic priming of gene regulatory elements is linked to cellular plasticity (Stadhouders et al., 2018). We therefore performed an assay for transposase-accessible chromatin with high-throughput sequencing (ATAC-seq; Buenrostro et al., 2013) to map genome-wide chromatin accessibility in KLRG1^+^ ILCs and ILC2s. We reproducibly detected 26,192 accessible regions across both cell types of which 273 were uniquely or substantially more (>2.0-fold) active in ILC2s and 474 in KLRG1^+^ ILCs (Fig. 6, A–C). Whereas many ILC2 signature genes, including *RORA*, *GFI1*, and *Bcl11b*, were associated with active chromatin both in ILC2s and KLRG1^+^ ILCs (Fig. S4), genes typically associated with ILC2 functionality, including the T helper type 2 cell (Th2 cell) cytokine locus, *IL9*, and *GATA3*, had an active chromatin signature specific for ILC2s (Fig. 6, D–F; and Fig. S4). Other enriched biological pathways in ILC2s included prostaglandin metabolism (*ANXA1, FABP5, HPGD, HPGDS*, and *PNPLA8*, in concordance with elevated *HPGDS* expression; Fig. 3 D) and T cell costimulation (e.g., *ICOS* and *ICOSL*; Fig. 6, D–F and Fig. S4). We found that gene regulatory elements bearing an active chromatin signature specific for KLRG1^+^ ILCs were located near genes enriched for processes involved in the differentiation of Th1, Th2, and Th17 cells (including *IL23R*, *IL12RB1/2*, and *IFNG*). These findings support the immature nature of the KLRG1^+^ ILCs and confirm our in vitro differentiation assays. KLRG1^+^ ILCs also showed regulatory activity near (TF) genes important for ILC development, such as *TCF7* and *PDCD1*. As expected, the *KLRG1* locus harbored accessible chromatin in both ILC2s and KLRG1^+^ ILCs. In summary, our regulome data are consistent with the data of the in vitro culture assays indicating that KLRG1^+^ ILCs are immature and able to develop into multiple ILC subsets.

### Functionality of KLRG1^+^ILCs and NKp46^+^ILCs in tissues

**Figure 7.**
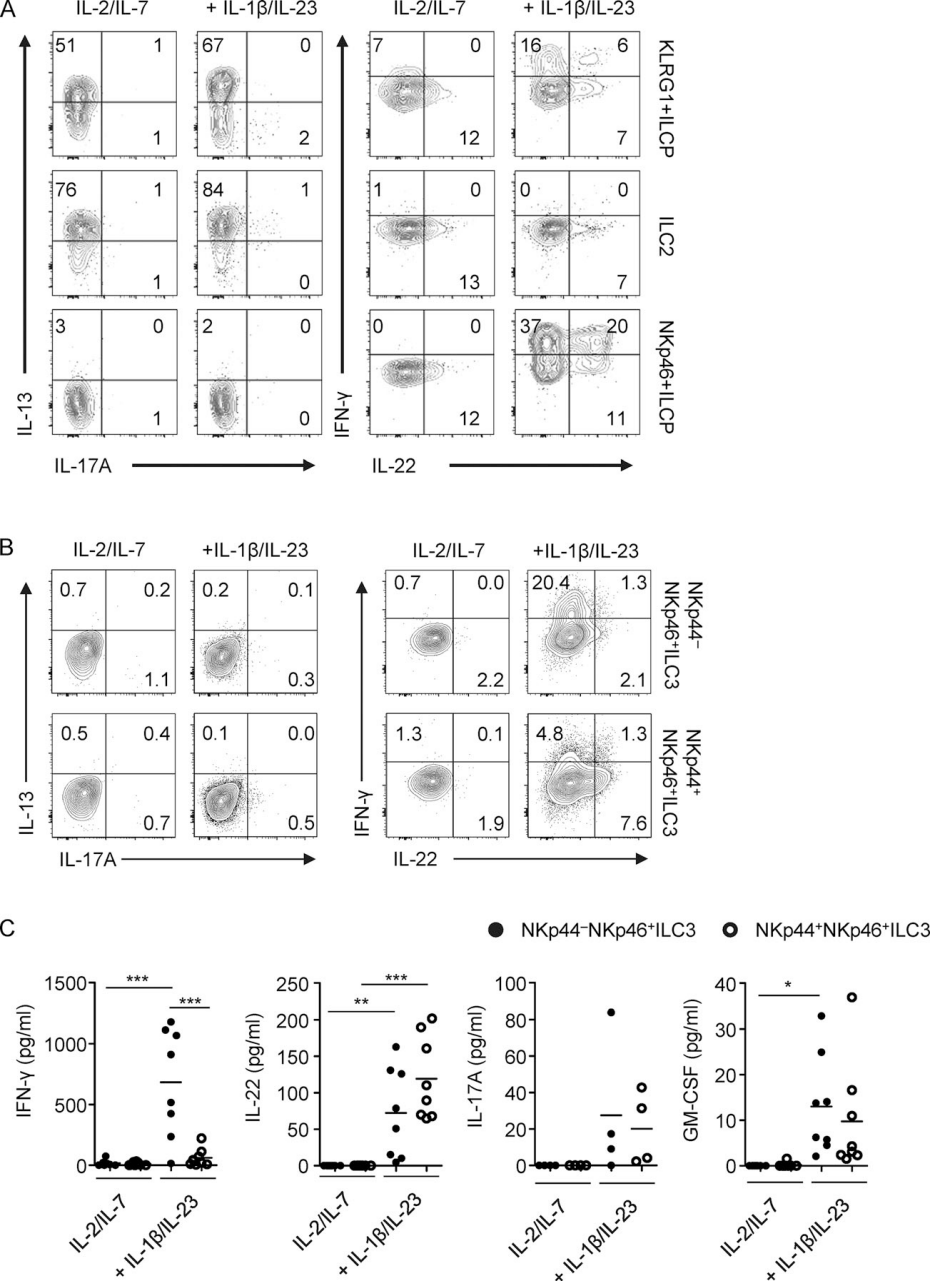
**Functionality of KLRG1^+^ ILCs and NKp46^+^ ILCs in tissues. (A)** Representative flow cytometric analysis of intracellular IL-13, IL-17A, IFN-γ, and IL-22 in KLRG1**^+^** ILCs, ILC2s, and NKp46**^+^** ILCs isolated from NPs, cultured on OP9-DL1 cells in the presence of IL-2 (20 U/ml) and IL-7 (20 ng/ml) with or without IL-1β and IL-23 (20 ng/ml each) for 7 d, and subsequently stimulated by PMA/ionomycin for 3 h. **(B)** Representative flow cytometric analysis of intracellular IL-13, IL-17A, IFN-γ, and IL-22 in NKp44^−^NKp46**^+^** ILC3s and NKp44**^+^**NKp46**^+^** ILC3s isolated from tonsils, cultured and stimulated as in A. **(C)** Quantification of cytokine production by ELISA in culture supernatants from cells stimulated as in A. The concentration is adjusted to 5,000 cells. Data in A and B are representative of at least three donors from three independent experiments.

As we identified populations phenotypically similar to KLRG1^+^ ILCs and NKp46^+^ ILCs in different tissues, we studied the cytokine potential of these tissue-derived ILCs. Culture of KLRG1^+^ ILCs and ILC2s from NPs from CRS patients on OP9-DL1 cells resulted in IL-13–producing cells (Fig. 7 A). In response to IL-1β and IL-23, KLRG1^+^ ILCs clearly produced IFN-γ and low amounts of IL-22, whereas NP ILC2s did not. In contrast to KLRG1^+^ ILCs from blood, we hardly detected production of IL-17A by NP KLRG1^+^ ILCs. NKp46^+^ ILCs from this tissue were not able to produce either IL-13 or IL-17A but were able to produce IL-22 and IFN-γ in response to IL-1β and IL-23 (Fig. 7 A).

Tonsil ILC3s are very distinct from PB NKp46^+^ ILCs, and in response to IL-1β and IL-23, these cells were more potent IL-22 producers than PB NKp46^+^ ILCs. Tonsil ILC3s also produced low levels of GM-CSF and IL-17 (Fig. 7, B and C). Interestingly, whereas NKp44^−^ ILC3 from tonsils produced high amounts of IFN-γ, tonsillar NKp44^+^ ILC3s were poor IFN-γ producers (Fig. 7, B and C). It should be noted that ex vivo–isolated tonsillar NKp44^−^ ILC3s did not produce IFN-γ, IL-22, and IL-17A, whereas NKp44^+^ ILC3s produced IL-22, but not IFN-γ or IL-17A (Fig. S5, A and B). In conclusion, this shows that tissues contain cells phenotypically similar to circulating KLRG1^+^ and NKp46^+^ ILCs, but these cells have probably already been imprinted in the tissue and are more restricted to the ILC2 and ILC3 fate, respectively.

## Discussion

Recently, PB and tonsil CD34^−^ CD117^+^ ILCs were described to enclose cells that have the capacity to develop into multiple ILC subsets (Lim et al., 2017; Chen et al., 2018). Our data indicate that the CD117^+^ ILC population is heterogeneous and contains ILCs that belong to either the ILC2 or ILC3 lineage. HSNE analysis revealed the presence of PB ILCs that express KLRG1, known to be present both on mouse (Hoyler et al., 2012) and human ILC2s (Salimi et al., 2013; Simoni et al., 2017), but lack the human ILC2 marker CRTH2. We propose that these KLRG1^+^ILCs are precursors of ILC2s, because they are transcriptionally similar to CRTH2^+^ ILC2s but express relatively low levels of cytokine receptors and genes involved in ILC2 functionality. KLRG1^+^ ILCs express less *GATA3* and its putative targets *HPGDS* and *FCRL3* than ILC2s and have less accessible chromatin regions that are associated with ILC2-specific genes. Importantly these cells can differentiate into cytokine-producing CRTH2^+^ ILC2s in vitro.

Whereas KLRG1^+^ ILCs and ILC2s produce similar amounts of IL-5 and IL-13 upon activation with TSLP and IL-33, a proportion of KLRG1^+^ ILCs could acquire the capacity to produce IL-17A, IL-22, and IFN-γ when cultured with IL-1β and IL-23. This is similar to the ILC precursors described by Lim et al. (2017). These researchers demonstrated that CD117^+^ PB ILCs contain clones that are able to produce multiple cytokines upon culture with IL-2, IL-7, IL-1β, and IL-23. We showed that among those CD117^+^ PB ILCs, only KLRG1^+^ ILCs and KLRG1^−^ NKp46^−^ ILCs have this capacity. As the KLRG1^+^ ILCs produced type 2 cytokines, but not type 3 cytokines, upon culture with IL-33 and TSLP, the differentiation capacity of this subset depends on the kind of stimulus. Our findings therefore strongly suggest that the KLRG1^+^ ILCs are transitional cells that are biased to, but not yet fully committed to, the ILC2 fate. Indeed, our ATAC-seq analysis of KLRG1^+^ ILCs revealed epigenetic priming at loci associated with functionally different ILC fates, providing a molecular mechanism for the observed differentiation of KLRG1^+^ ILCs.

KLRG1 is also expressed by mouse ILC2s, although high expression was only observed after stimulation with IL-25 or upon *Nippostrongylus brasiliensis* infection. These mouse KLRG1^hi^ ILC2s were called inflammatory (iILC2s) and appeared to play an important role in the host defense during infection. iILC2s were shown to be highly migratory cells as compared with IL-33–responsive KLRG1^dim^ natural ILC2s. Interestingly, mouse iILC2s were multipotential and are able to differentiate into IL-17–producing ILC3-like cells and natural ILC2s (Huang et al., 2015, 2018). The KLRG1^+^ ILCs described here bear similarities with mouse iILC2s, as they express KLRG1 and can acquire the capacity of producing IL-17.

We also observed that the PB ILC population can be further segregated into three populations based on their expression of NKp46 and CD56. NKp46^+^ ILCs were able to differentiate into IL-22–producing ILC3s upon culture with OP9-DL1, IL-1β, and IL-23. The NKp46^+^ ILCs do not have the capacity to differentiate into ILC2s, suggesting that NKp46^+^ ILCs are comparable to the CD56^+^ NKp46^+^ ILCs described by Chen et al. (2018). The PB NKp46^+^ ILCs do not express cytokines when isolated ex vivo and share similarities with NKp44^−^ ILC3s isolated from resting secondary lymphoid organs (SLOs), except that the latter cells express more RORγt and IL23R than the former (Bar-Ephraim et al., 2017). Both PB NKp46^+^ ILCs and NKp44^−^ SLO ILC3s expressed higher migratory related genes such as *S1PR1* (Bar-Ephraim et al., 2017). However, NKp44^−^ ILC3s from SLOs are capable of producing cytokines upon IL-1β and IL-23 stimulation without Notch signaling (Bar-Ephraim et al., 2017). The notion that RORγt was only induced in NKp46^+^ ILCs upon Notch signaling suggests that peripheral S1PR1-expressing NKp46^+^ ILCs may migrate into tissues and require signals from the tissue microenvironment to become functional ILC3s. Freshly isolated PB NKp46^+^ ILCs expressed high levels of GATA3, and although this TF is associated with ILC2 development and function, it is also required for ILC3 development (Serafini et al., 2014; Zhong et al., 2016). The high *GATA3* levels may be responsible for the low *RORC* expression in NKp46^+^ ILCs as compared with NKp44^+^ ILC3s, as it is known that RORγt is negatively regulated by GATA3 at an early stage of development (Zhong et al., 2016). As the frequency of IL-17–producing NKp46^+^ ILCs is low, we conclude that they are biased mainly toward IL-22. These in vitro–differentiated ILC3s are therefore very similar to the mouse CCR6^−^ NKp46^+^ ILC3s that in response to IL-23 are potent IL-22– and IFN-γ–producing ILC3s but lack IL-17 potential (Klose et al., 2013).

Some of the NKp46^+^ ILCs in PB coexpressed CD56. These cells are most likely not NK cells, because they express CD127 and CD117 and lack CD16 and CD94 expression. Moreover, the CD56^+^ cells expressed CD200R, which was recently found to be expressed on mouse ILC1s, but not on NK cells (Weizman et al., 2017). Human ILC2s also express CD200R (Blom et al., 2017), and here, we show that this marker is expressed on all ILC subsets. The CD56^+^NKp46^+^ ILCs are most likely derived from NKp46^+^ ILCs, because these ILCs up-regulated CD56 in the differentiation cultures and CD56^+^ ILCs could also give rise to IL-22– and IFN-γ–producing cells (Chen et al., 2018). This is consistent with a previous report on CD56 expression on IL-22 producing ILC3s (Cella et al., 2009). As reported earlier (Lim et al., 2017; Chen et al., 2018), there is a link between NKp46^+^ ILCs and NK cells, because both NKp46^+^ ILCs and ILC3s seem to possess the potential to differentiate into NK cells, particularly when cultured with IL-12 or IL-15 (Kyoizumi et al., 2017; Raykova et al., 2017). Whether this means that these NKp46^+^ ILCs represent an obligatory transitional cell type in NK cell development remains unclear. It should, however, be noted that the NKp46^+^ ILCs expressed high levels of CD117, and because CD117^high^ ILCs are absent in *RORC*-deficient patients (Okada et al., 2015), it is likely that NKp46^+^ ILCs are absent in these patients. In contrast, NK cell numbers in these patients are in the normal range, strongly suggesting that NK cell development can proceed in the absence of NKp46^+^ ILCs. It is, however, possible that NK cell development via NKp46^+^ ILCs is an alternative pathway. It is in this context noteworthy that KLRG1^+^ ILCs and ILC2s also differentiated into EOMES and CD94-expressing NK-like cells and ILC1s in the presence of IL-1β and IL-12 (Bal et al., 2016; Silver et al., 2016).

As stated above, CD117^high^ ILCs are absent in patients with deficiencies in *RORC* (Okada et al., 2015). Although we were unable to verify this, we speculate that the CD117^dim^ cells, which are still present in *RORC*-deficient patients, are identical to the KLRG1^+^CD117^+^ ILCs identified here, since Lim et al. (2017) observed that the CD117^dim^ cells from *RORC*-deficient patients can develop into ILCs that can produce both type 2 cytokines and the type 3 cytokine IL-22 (but not IL-17, which requires RORγt) when cultured on OP9-DL with IL-2, IL-7, IL-1β, and IL-23 (Lim et al., 2017). These data are consistent with our observation that KLRG1^+^ ILCs, while preferentially developing into ILC2s, can under inflammatory conditions also differentiate into ILCs with a broader cytokine secretion profile. We show that the remaining CD117^+^NKp46^−^KLRG1^−^IL1R1^+/−^ population, which represents ∼5% of the CD117^+^ ILC population in the PB, have the capacity to develop to all ILCs. Importantly, these cells lack CD34, which is expressed on hematopoietic stem cells and committed precursors. For instance, human pre-T cells, (pre)pro-B cells, and myeloid precursors all express CD34. Indeed, committed ILC3 precursors and common ILC/NK precursors identified in human SLOs and intestinal lamina propria express CD34 (Montaldo et al., 2014; Scoville et al., 2016). It still remains to be studied how CD34^+^ ILCs give rise to the circulating CD117^+^ ILC subsets that eventually develop into mature ILCs.

Collectively, our results indicate that KLRG1^+^ ILCs are transitional cells that are biased to differentiate into ILC2s but under inflammatory conditions are able to produce multiple ILC signature cytokines. Moreover, NKp46 clearly defines ILC3-lineage–biased cells that retained the capacity to differentiate into ILC1s in the presence of IL-12, like KLRG1^+^ ILCs, but are unable to develop into ILC2s.

## Materials and methods

### Human blood and tissue samples

Buffy coats were provided by the blood bank (Sanquin, Amsterdam). Tonsils were obtained from routine tonsillectomies, and tissue collection was done at the Academic Medical Center (AMC), Onze Lieve Vrouwe Gasthuis Hospital (Amsterdam, The Netherlands). Inflamed NPs were obtained from CRS patients during surgery, and umbilical cord blood was collected at the AMC. The collection and use of all human samples was approved by the Medical Ethical Committee of the AMC and with informed consent.

### Isolation of ILCs from blood and tissues

Peripheral blood mononuclear cells were isolated by Ficoll-Hypaque density gradient (Lymphoprep; Axis-Shield). Tonsil tissue was cut in fine pieces and mechanically disrupted using the Stomacher 80 Biomaster. Cell suspensions were filtered through a 70-µm cell strainer. Nasal tissues were manually cut into fine pieces and digested for 45 min at 37°C with Liberase TM (125 µg/ml) and DNase I (50 U/ml). Cell suspensions were filtered through a 70-µm cell strainer. Mononuclear cells were then isolated by Ficoll-Hypaque density gradient. Subsequently, ILCs were isolated as described previously (Krabbendam et al., 2018). Briefly, PB and cord blood were enriched for ILCs by immunomagnetic cell sorting using a negative selection of CD3, CD14, CD16, and CD19 with the Mojosort magnetic cell separation system (BioLegend). Tonsillar mononuclear cells were depleted of CD3 and CD19 cells by magnetic cell sorting (MACS) depletion. The cell suspensions were stained with antibodies against lineage (CD1a, CD3, CD4, CD5, CD14, CD19, CD16, CD34, CD94, CD123, BDCA2, TCRαβ, TCRγδ, and FcER1α), CD45, CD161, CD127, CD117, CRTH2, KLRG1, NKp44, NKp46, CD56, and IL-1R1. Detailed information about antibodies and other reagents can be found in Table S2. Cells were sorted on a FACSAria, and purity of the sorted cells in all experiments was >99%.

### Flow cytometry analysis

For experiments involving intracellular cytokine staining, cells were stimulated with PMA (10 ng/ml; Sigma) plus Ionomycin (500 nM; Merck) in the presence of Golgi Plug (BD Biosciences) for 3 h at 37°C. For FACS analysis, cultured cells were washed with PBS and stained with fixable viability dye eFluor 455UV (eBioscience) for 30 min at 4°C in PBS, followed by cell-surface antigen staining with antibodies for 20 min at 4°C in PBS. Then cells were fixed and permeabilized using the Foxp3/Transcription Factor Staining Buffer Kit (Thermo Fisher Scientific), and intracellular cytokines were stained with antibodies for 30 min at room temperature in permeabilization buffer. Samples were acquired on LSRFortessa or FACSCanto II (BD Biosciences) and analyzed with FlowJo software and Cytosplore^+HSNE^ (van Unen et al., 2017).

### Cell lines and ILC bulk and single-cell co-culture with OP9 cells

The naive OP9 murine stromal cell line was kindly provided by Dr. T. Nakano (Osaka University, Osaka, Japan). OP9-DL1 cells were generated as previously described (Dontje et al., 2006). Ex vivo–isolated ILCs were co-cultured with OP9 or OP9-DL1 in Yssel’s medium (IMDM supplemented with 4% [vol/vol] Yssel’s supplement (made in-house; AMC) and 2% [vol/vol] human AB serum [Invitrogen]). OP9 or OP9-DL1 cells (3,000 cells in 100 µl Yssel’s medium per well in 96-well round-bottom plates) were plated 1 d before co-culture. Bulk ILCs (100–1,000 cells) were cultured for 5–10 d with combinations of IL-2 (20 U/ml), IL-7, IL-33, TSLP, IL-1β, IL-23 (all at a concentration of 20 ng/ml), and cytokines were supplemented on day 5. For single-cell cloning experiments, cytokines and medium were replenished once per week, and cells were analyzed after 2–3 wk.

### Microarray analysis

To isolate total RNA, sorted cells were flash frozen in PBS immediately after sorting and stored at −80°C before RNA extraction. QIAzol Lysis Reagent (Qiagen) was added to the cells, and RNA was isolated and purified using the RNeasy kit (Qiagen). The concentration was measured on a NanoDrop ND-2000 (Thermo Fisher Scientific), and RNA integrity was examined using the 2200 TapeStation System with Agilent RNA ScreenTapes (Agilent Technologies). Total RNA was amplified using the GeneChip WT Pico Kit (Thermo Fisher Scientific) generating biotinylated sense-strand DNA targets. The labeled samples were hybridized to human Clariom S pico arrays (Thermo Fisher Scientific). Washing and staining was performed using the GeneChip Fluidics Station 450, and scanning was performed using the GeneChip Scanner 3000 (both Thermo Fisher Scientific). All cell populations were generated in triplicate. All data analysis was performed in RStudio. Raw data were normalized using the robust multi-array average (RMA) algorithm implemented in the limma Bioconductor R-package (Ritchie et al., 2015). Adjusted P values were calculated using the Benjamini–Hochberg method. Data were visualized using glimma and pheatmap R packages (Su et al., 2017). The R package UpsetR was used to visualize cloning experiments (Lex et al., 2014).

### Quantitative real-time PCR

Total RNA was extracted with a NucleoSpin RNA XS kit (Macherey-Nagel) according to the manufacturer's instructions. cDNA was synthesized with a High-Capacity cDNA Archive kit (Applied Biosystems). PCRs were performed in a Bio-Rad iCycler with IQ SYBR Green Supermix (Bio-Rad). The primer sets used are listed in Table S2. Bio-Rad CFX Manager 3.1 software was used for quantification of expression. All samples were normalized to the expression of GAPDH, and results are presented in arbitrary units.

### ATAC-seq

ATAC-seq was performed as previously described (Buenrostro et al., 2013) with minor modifications. Cells from three donors were sorted (as described above; 20,000 ILC2s and ∼2,000 KLRG1^+^ILCs per donor) in freshly prepared cold ATAC-seq lysis buffer (10 mM Tris-HCl, pH 7.5, 60 mM KCl, 15 mM NaCl, 5 mM MgCl, 0.1 mM EGTA, 0.3 M sucrose, 0.1% NP-40, 0.15 mM spermine, 0.5 mM spermidine, and 2 mM 6AA). After sorting was completed, lysis buffer was added to 1 ml, and samples were centrifuged for 10 min at 500 ×*g* (4°C) and further processed as originally described (Buenrostro et al., 2013). ATAC-seq libraries were sequenced on an Illumina HiSeq2500 (paired-end read mode, 51-bp read length), and reads were aligned to the human genome (hg38 build) using HISAT2 (Kim et al., 2015). Paired alignments spanning >1,000 bp were filtered from the alignments, and MACS2 software was used to call peaks. Overlapping and nonoverlapping regions/peaks between two samples were identified using the intersect function of BEDTools (Quinlan and Hall, 2010) or the HOMER (Heinz et al., 2010) *mergePeaks* script (-d given option) requiring a minimal overlap of 1 bp. Only peaks found in at least two out of three replicates were kept for downstream analysis. The HOMER *annotatePeaks* script was then used to quantify normalized read densities for all reproducible peaks (*n* = 26,192) in each replicate dataset. Differentially enriched peaks were selected based on changes in normalized average read density (>2.0 fold) and statistical significance (P < 0.05, paired *t* test), after which we removed low signal peaks (<3.0 average log2 normalized read density in both ILC subsets). Gene-peak associations were obtained using the HOMER *annotatePeaks* script; pathway enrichment analysis was performed using Metascape (Tripathi et al., 2015). PCA and Ward hierarchical clustering were performed in R using RStudio (v1.1.463).

### Statistical analysis

Data are represented as individual values with mean unless specified. Sample size for each experiment and the replicate number of experiments are included in the figure legends.

### Data and software availability

The data reported in this paper have been uploaded to the Gene Expression Omnibus with the accession numbers GSE123817 and GSE124054.

### Contact for reagent and resource sharing

Further information and requests for resources and reagents should be directed to and will be fulfilled by corresponding author Hergen Spits.

### Online supplemental material

Fig. S1 shows further HSNE analysis of cluster B as identified by HSNE analysis of total PB ILCs; CD200R expression on ILC subsets and NK cells; HSNE analysis of total tonsil ILCs; ILC distribution in PB, tonsils, and NPs; and the frequency of KLRG1^+^ ILCs in these tissues. Fig. S2 shows the phenotype of PB KLRG1^+^ ILCs, NKp46^+^ ILCs, ILC2s, and tonsil NKp44^−^ and NKp44^+^ ILC3s after culture on OP9-DL1 cells with IL-2 and IL-7 and relative expression of *RORC*, *EOMES*, *TBX21*, *GZMB*, and *PRF1* in different ILC subsets isolated from PB and tonsils. Fig. S3 shows the TF expression in different KLRG1^+^ and NKp46^+^ ILC clones and the cytokine production from bulk and clonal differentiation cultures of NKp46^−^ ILCs. Fig. S4 shows additional genome browser shots of ATAC-seq signals across key loci relevant for ILC development and differentiation. Fig. S5 shows the cytokine production profile of freshly isolated NKp44^−^ and NKp44^+^ ILC3s from tonsils.

## Supplementary Material

Supplemental Materials (PDF)

Table S1 (Excel file)
