# Correction: KLRG1 and NKp46 discriminate subpopulations of human CD117^+^CRTH2^−^ ILCs biased toward ILC2 or ILC3

**DOI:** 10.1084/jem.2019049007302019c

**Published:** 2019-08-06

**Authors:** Maho Nagasawa, Balthasar A. Heesters, Chantal M.A. Kradolfer, Lisette Krabbendam, Itziar Martinez-Gonzalez, Marjolein J.W. de Bruijn, Korneliusz Golebski, Rudi W. Hendriks, Ralph Stadhouders, Hergen Spits, Suzanne M. Bal

Vol. 216, No. 8, August 5, 2019. 10.1084/jem.20190490.

*JEM* regrets that in the original version of this paper, panels E-G were mistakenly omitted from Fig. 4 due to a production error. The corrected and complete Fig. 4 appears on the following page.

**Figure 4.**
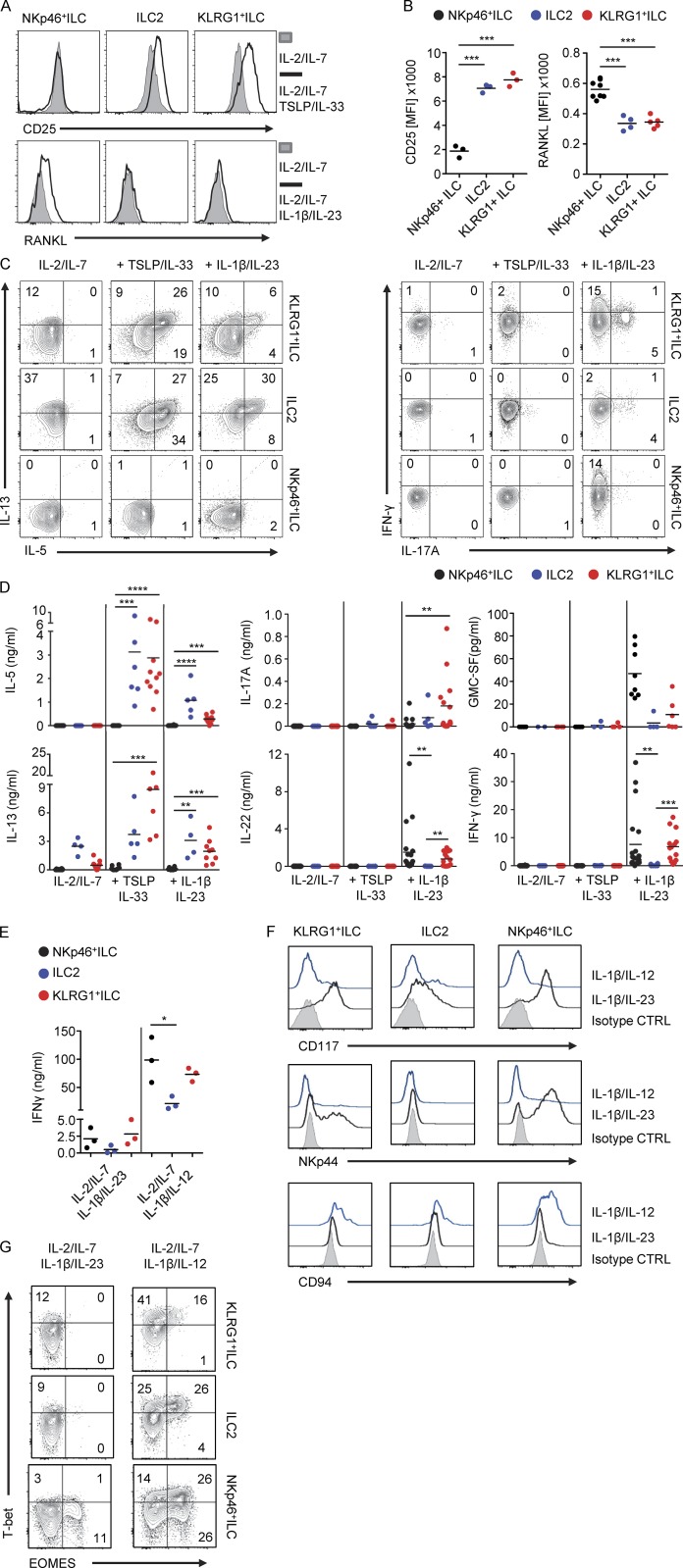
**KLRG1**^**+**^ **ILCs and NKp46**^**+**^ **ILCs show multipotent cytokine production and TF profile. (A)** Representative histogram of expression of CD25 or RANKL on KLRG1^+^ ILCs, ILC2s, and NKp46^+^ ILCs after culture on OP9-DL1 cells in the presence of IL-2 and IL-7 with or without TSLP and IL-33 or IL-1β and IL-23 for 7 d. **(B)** Quantification of CD25 or RANKL expression on after culture as in A (*n* = 3–8). **(C)** Representative flow cytometric analysis of intracellular IL-5, IL-13, IFN-γ, and IL-17A in KLRG1^+^ ILCs, ILC2s, and NKp46^+^ ILCs, after 7 d culture as in A and subsequently stimulated by PMA/ionomycin for 3 h. **(D)** Quantification of cytokine production by ELISA in culture supernatants from cells stimulated as in A. The concentration is adjusted to 5,000 cells. **(E)** Quantification of IFN-γ production by ELISA of ILC subsets cultured on OP9-DL1 cells in the presence of IL-2 and IL-7 with IL-1β and IL-23 or IL-1β and IL-12 for 7 d. **(F)** Representative flow cytometry of the expression of CD117, NKp44, and CD94 on KLRG1^+^ ILCs, ILC2, and NKp46^+^ ILCs after culture as in E. Filled histograms represent isotype control. **(G)** Representative flow cytometric analysis of intracellular expression of EOMES and T-bet in KLRG1^+^ ILCs, ILC2s, and NKp46^+^ ILCs after culture as in E. Data in A, C, F, and G are representative of at least three donors from more than three independent experiments. Cytokines used in all experiments are IL-2 (20 U/ml), IL-7, TSLP, IL-33, IL-1β, IL-23, and IL-12 (all 20 ng/ml). **, P < 0.001; ***, P < 0.0001; ****, P < 0.00001 (one-way ANOVA).

The online and print versions of this article have been corrected. The error appears only in PDF versions downloaded on or before July 31, 2019.

