# Supplementary material for: KLRG1 and NKp46 discriminate subpopulations of human CD117+CRTH2− ILCs biased toward ILC2 or ILC3
Source: J Exp Med. 2019 Jun 14;216(8):1762–76. doi: 10.1084/jem.20190490 (PMC6683990; doi:10.1084/jem.20190490)
Supplement: Supplemental Materials (PDF) [file JEM_20190490_sm.pdf]

Supplemental material

Nagasawa et al., https://doi.org/10.1084/jem.20190490

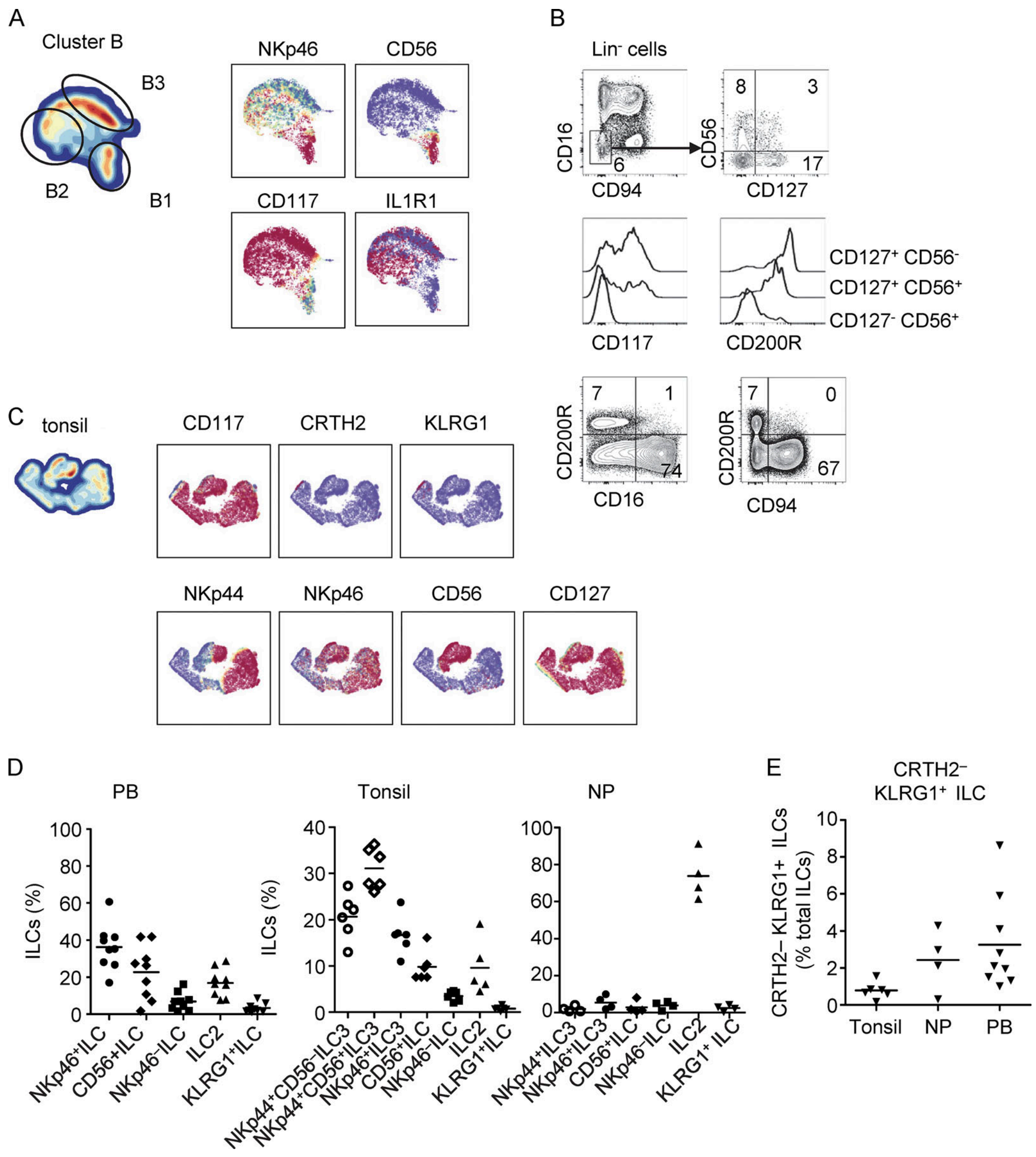

Figure S1. **Characterization of ILCs in PB, tonsil, and NP. (A)** HSNE analysis of zoom-in of ILC cluster B (from Fig. 1 A; *n* = 8). **(B)** Expression of CD127 and CD56 in PB Lin$^-$CD94$^-$CD16$^-$ cells and expression of CD117 and CD200R in CD127$^+$CD56$^-$, CD127$^+$CD56$^+$, and CD127$^-$CD56$^+$ cells (*n* = 6). **(C)** HSNE analysis of the ILC population in tonsil (*n* = 4). **(D)** Frequency of each ILC subset within PB, tonsil, and NP. **(E)** Frequency of CRTH2$^-$ KLRG1$^+$ ILCs within tonsil, NP, and PB. All data are verified in at least two independent experiments.

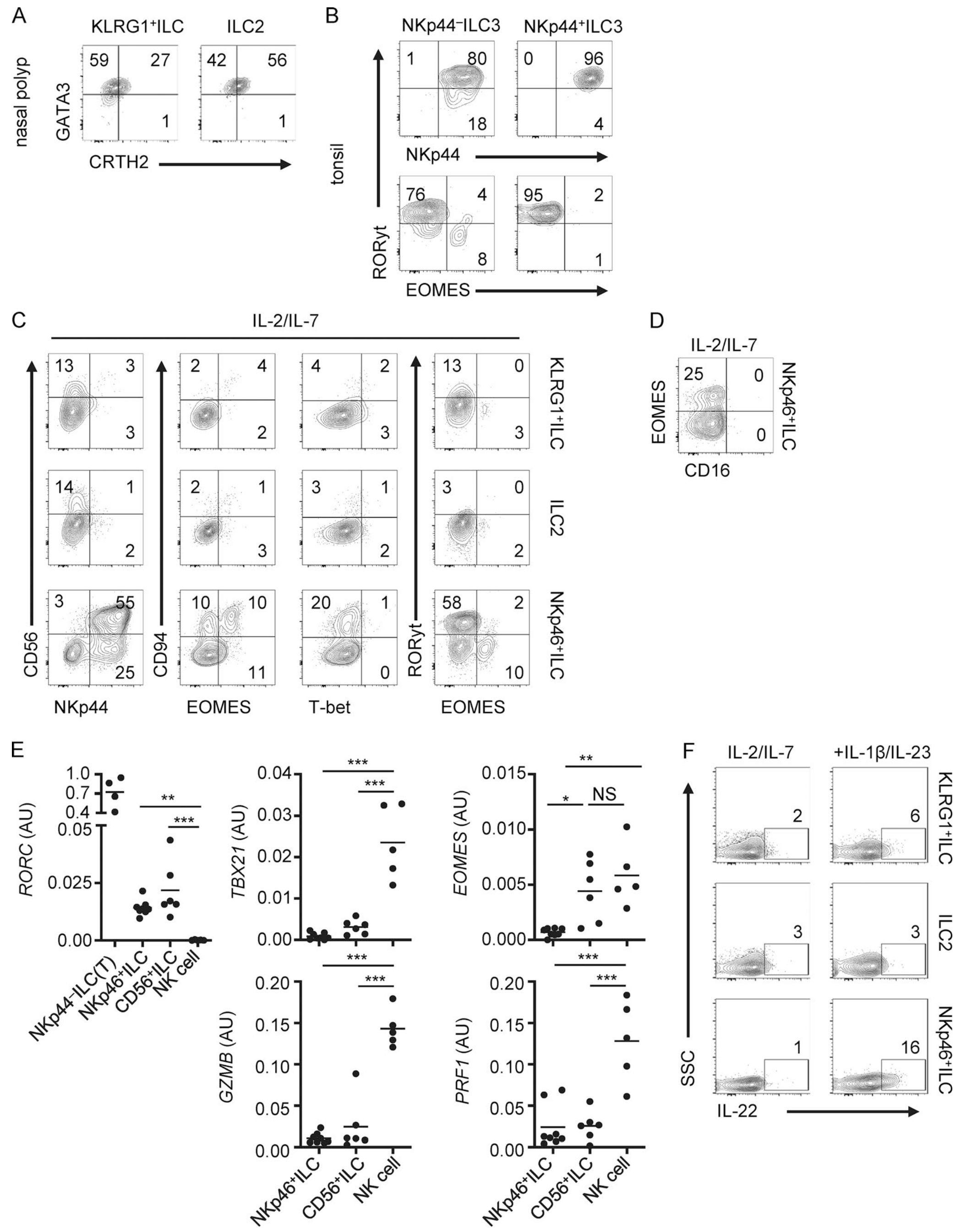

Figure S2. **Differentiation capacity of KLRG1$^+$ ILCs and NKp46$^+$ ILCs. (A and B)** Expression of CRTH2 and GATA3 in KLRG1$^+$ ILCs and ILC2s isolated from NP (n = 3; A) NKp44, RORγt, and EOMES on NKp44$^-$ ILC3s and NKp44$^+$ ILC3s (both NKp46$^+$ CD56$^-$) isolated from tonsils (B) after culture for 7 d on OP9-DL1 in the presence of IL-2 (20 U/ml) and IL-7 (20 ng/ml; n = 3). **(C)** Expression of CD56, NKp44, RORγt, and EOMES in PB isolated ILC subsets after culture for 5 d on OP9-DL1 in the presence of IL-2 and IL-7 (n = 6). **(D)** Expression of EOMES and CD16 in PB isolated NKp46$^+$ ILCs after cultured as in C (n = 3). **(E)** Quantification of relative RORC, EOMES, TBX21, GZMB, and PRF1 expression as compared with GAPDH in different ILC subsets isolated from tonsil (T) and PB. Each dot represents one donor. **(F)** Intracellular IL-22 expression after culture for 7 d on OP9-DL1 in the presence of IL-2, IL-7, IL-1β, and IL-23 (n = 3). *, $P < 0.05$; **, $P < 0.001$; ***, $P < 0.0001$ (one-way ANOVA). All data are verified in at least two independent experiments. AU, area under the curve. SSC, side scatter.

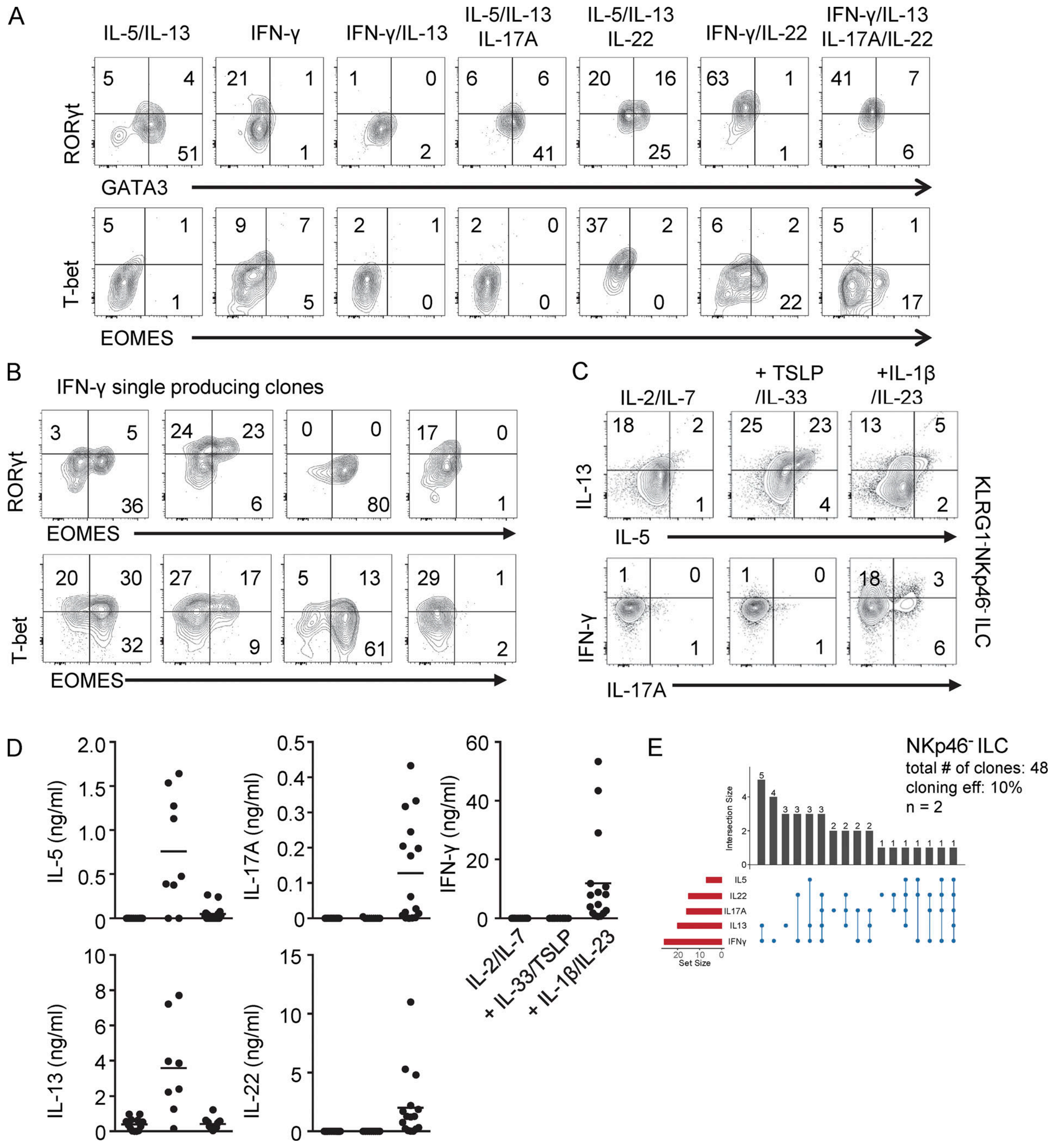

Figure S3. **Clonal analysis of CD117⁺ PB ILC subsets show broad functional differentiation capacities. (A)** Representative flow cytometric analysis of intracellular GATA3, RORγt, T-bet, and EOMES expression in clones obtained from KLRG1⁺ ILCs cultured for 14–21 d on OP9-DL1 with IL-2 (20 U/ml), IL-7, IL-1β, and IL-23 (20 ng/ml each). **(B)** Representative flow cytometric analysis of NKp46⁺ ILC clones producing IFN-γ and their TF expression profile. **(C)** Representative flow cytometric analysis of intracellular IL-5, IL-13, IFN-γ, and IL-17A in KLRG1⁻NKp46⁻ ILCs after 7 d cultured on OP9-DL1 cells with IL-2 and IL-7 with or without TSLP and IL-33 or IL-1β and IL-23. **(D)** Quantification of cytokine production by ELISA in culture supernatants from cells stimulated as in C. The concentration is adjusted to 5,000 cells. **(E)** Summary of numbers and type of cytokines produced by NKp46⁻ ILC clones. Data in A–C are representative of at least three donors from two independent experiments.

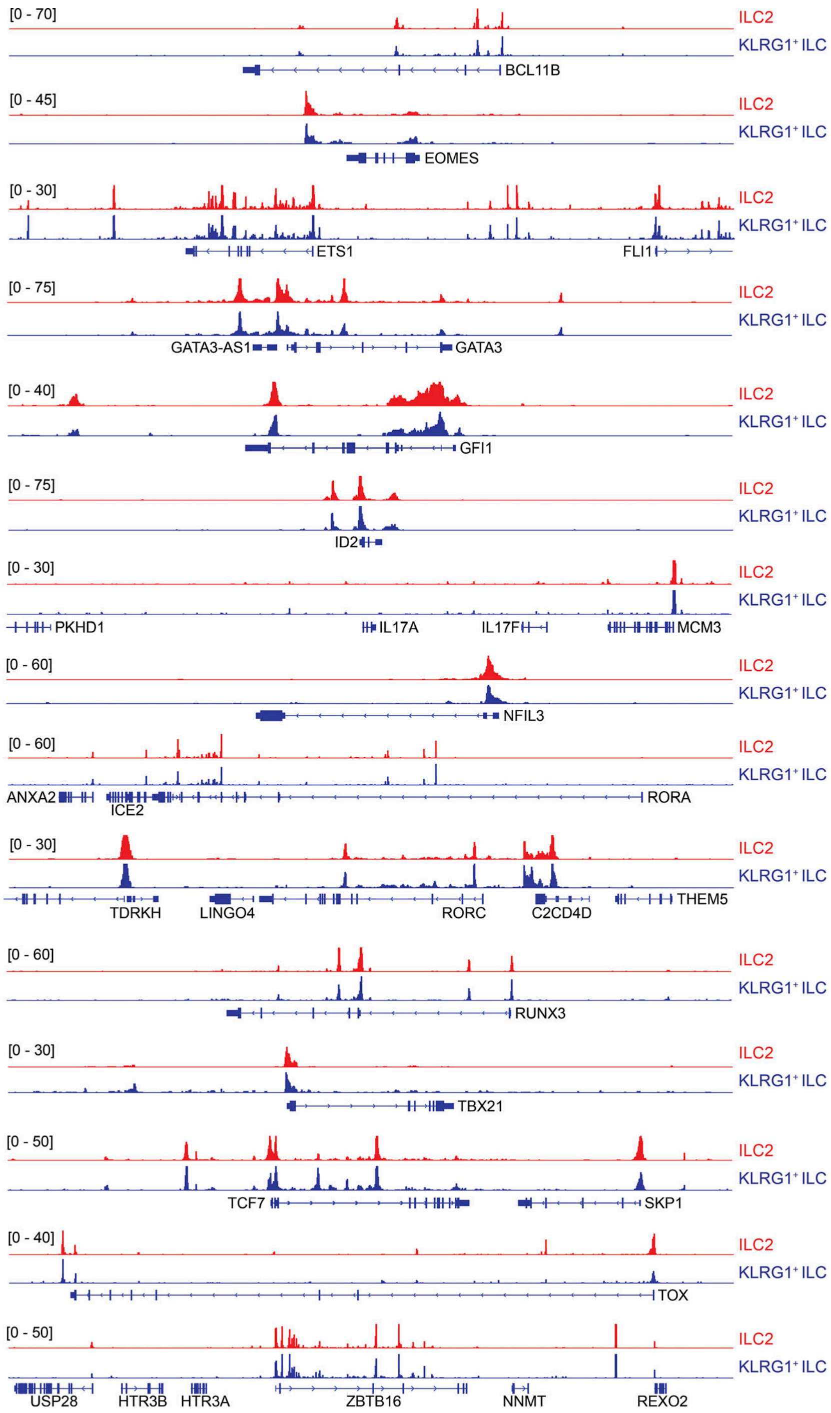

Figure S4. **Chromatin accessibility as measured by ATAC-Seq at key loci related to ILC biology.** Additional genome browser shots of ATAC-Seq signals across key loci relevant for ILC development and differentiation.

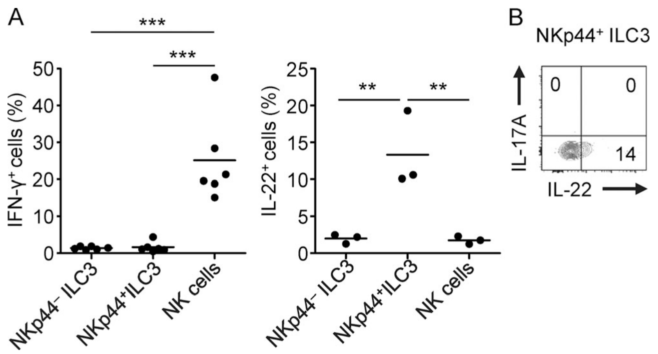

Figure S5. **Cytokine production profile of freshly isolated NKp44⁻ ILC3 and NKp44⁺ ILC3 from tonsil. (A)** IFN-γ and IL-22 production by freshly isolated NKp44⁻ ILC3s and NKp44⁺ ILC3 from tonsils were evaluated by intracellular cytokine staining after stimulation with PMA/ionomycin for 6 h. NK cells were used as a reference. Each dot represents an individual donor. **(B)** Representative flow cytometry plot of IL-17A and IL-22 production by freshly isolated NKp44⁺ ILC3s after stimulation with PMA/ionomycin for 6 h (n = 6). **, P < 0.001; ***, P < 0.0001 (one-way ANOVA).

Table S2. **Key resources**

Reagent or resource	Source	Identifier
**Antibodies**		
anti-human CD1a FITC (HI149)	BioLegend	Cat#300104, RRID:AB_314018
anti-human CD3 FITC (OKT3)	BioLegend	Cat#317306, RRID:AB_571907
anti-human CD4 FITC (RPA-T4)	BioLegend	Cat#300506, RRID:AB_314074
anti-human CD5 FITC (UCHT2)	BioLegend	Cat#300606, RRID:AB_314092
anti-human CD14 FITC (HCD14)	BioLegend	Cat#325604, RRID:AB_830677
anti-human CD16 FITC (3G8)	BioLegend	Cat#555406, RRID:AB_395806
anti-human CD19 FITC (HIB19)	BioLegend	Cat#302206, RRID:AB_314236
anti-human CD34 FITC (581)	BioLegend	Cat#343504, RRID:AB_1731852
anti-human CD94 FITC (DX22)	BioLegend	Cat#305504, RRID:AB_314534
anti-human CD123 FITC (6H6)	BioLegend	Cat#306014, RRID:AB_2124259
anti-human FcER1α FITC (AER37)	BioLegend	Cat# 334608, RRID:AB_1227653
anti-human TCRαβ FITC (IP26)	BioLegend	Cat#306706, RRID:AB_314644
anti-human TCRγδ FITC (B1)	BioLegend	Cat#331208, RRID:AB_1575108
anti-human BDCA2 FITC (201A)	BioLegend	Cat#354208, RRID:AB_2561364
anti-human CD161 PE (HP-3G10)	BioLegend	Cat#339904, RRID:AB_1501083
anti-human NKp44 PE (P448)	BioLegend	Cat#325108, RRID:AB_756100
anti-human IL-5 PE (JES1-39D107)	BioLegend	Cat#500904, RRID:AB_315139
anti-human CD45 AF700 (HI30)	BioLegend	Cat#304024, RRID:AB_493761
anti-human CD3 AF700 (UCHT1)	BioLegend	Cat#300424, RRID:AB_493741
anti-human IL-17A AF700 (BL168)	BioLegend	Cat#512318, RRID:AB_2124868
anti-human CD161 BV421 (HP-3G10)	BioLegend	Cat#339914, RRID:AB_2561421
anti-human IL-5 BV421 (JES1-39D10)	BioLegend	Cat#504311, RRID:AB_2563161
anti-human IFNγ BV510 (4S.B3)	BioLegend	Cat#502544, RRID:AB_2563883
anti-human CRTH2 PE-CF594 (BM16)	BioLegend	Cat#350126, RRID:AB_2572053
anti-human IL-13 APC (JES10-5A2)	BioLegend	Cat#501903, RRID:AB_315198
Anti-human CD5 APC Cy7(L17F12)	BioLegend	Cat#364010, RRID:AB_2564506
Anti-human CD161 APC/Fire 750 (HP-3G10)	BioLegend	Cat#339944, RRID:AB_2617016
Anti-human NKp46 BV421 (9E2)	BioLegend	Cat#331914, RRID:AB_2563853
Anti-human T-bet BV605 (4B10)	BioLegend	Cat#644817, RRID:AB_11219388
Anti-human CCR6 BV605(G034E3)	BioLegend	Cat#353420, RRID:AB_2561449
Anti-human CD56 BV650 (HCD56)	BioLegend	Cat#318344, RRID:AB_2563838
Anti-human CD3 Biotin (OKT3)	BioLegend	Cat#317320, RRID:AB_10916519
Anti-human CD19 Biotin (HIB19)	BioLegend	Cat#302204, RRID:AB_314234
Anti-human CD14 Biotin (62D3)	BioLegend	Cat#367106, RRID:AB_2566618
Anti-human CD16 Biotin (3G8)	BioLegend	Cat#302004, RRID:AB_314204
anti-human T-bet PE-Cy7 (4B10)	eBioscience	Cat#12582582, RRID:AB_925761
anti-human GATA3 PE (TWAJ)	eBioscience	Cat#12996642, RRID:AB_1963600
anti-human KLRG1 APC (13F12F2)	eBioscience	Cat#17948842, RRID:AB_2573303
Anti-human IL-22 PE Cy7(22URT1)	eBioscience	Cat#25-722-42
Anti-human IL1R1 PE	eBioscience	Cat#FAB269p, RRID:AB_2124912
Anti-human EOMES PE-eFluor610(WD1928)	eBioscience	Cat#61-4877-42, RRID: AB_2574616
anti-human CD127 PE-Cy7 (R34.34)	Beckman Coulter	Cat#A64618

Table S2. **Key resources (*Continued*)**

Reagent or resource	Source	Identifier
anti-human CD117 PE-Cy5 (104D2D1)	Beckman Coulter	Cat# IM2733, RRID:AB_131178
anti-human CRTH2 AF647 (BM16)	Beckman Dickinson	Cat#558042, RRID:AB_2112699
anti-human CD45 APC-Cy7 (2D1)	Beckman Dickinson	Cat#368518, RRID:AB_2616705
anti-human RoRγT AF647 (Q21-559)	Beckman Dickinson	Cat#563620, RRID:AB_2738324
**Bacterial and virus strains**		
N/A		
**Biological samples**		
Healthy human peripheral blood	Sanquin Bloodbank Amsterdam	N/A
Human nasal tissue	Amsterdam UMC	N/A
Human tonsil tissue	Amsterdam UMC/OLVG hospital Amsterdam	N/A
Human AB serum	Merck	Cat#H4522
**Chemicals, peptides, and recombinant proteins**		
Recombinant human IL-1b	R&D Systems	Cat#201-LB
Recombinant human IL-2	R&D Systems	Cat#202-IL-500
Recombinant human IL-7	Peprotech	Cat# 200-07
Recombinant human IL-23	R&D Systems	Cat#1290-IL-010
Recombinant human IL-33	R&D Systems	Cat#3625-IL-010
Recombinant human TSLP	R&D Systems	Cat#1398-TS-010
Phorbol 12-Myristate 13-acetate (PMA)	Sigma-Aldrich	Cat#P8139
Ionomycin	Merck	Cat#407950
BD Golgiplug Protein Transport Inhibitor	BD Biosciences	Cat#555029
Iscove's Modified Dulbeco's Medium	Gibco	Cat#21980-065
Roswell Park Memorial Institute (RPMI)1640	Gibco	Cat#52400-041
Anti-PE microbeads	Miltenyi	Cat#130-048-801
Anti-FITC microbeads	Miltenyi	Cat#130-048-701
AutoMACS Running Buffer – MACS Separation Buffer	Miltenyi	Cat#130-091-221
MACS separation columns LS	Miltenyi	Cat#130-042-401
MACS separation columns LD	Miltenyi	Cat#130-042-901
Pre-Separation Filters (30 µm)	Miltenyi	Cat#130-041-407
MojoSort streptavidin Nanobeads	BioLegend	Cat#480016
Lymphoprep	Stemcell Technologies	Cat#07861
Liberase TM	Roche	Cat#5401127001
DNAse I	Roche	Cat#11284932001
Penicillin-Streptomycin	Roche	Cat#11074440001
HyClone™ FetalClone™ I Serum	ThermoFisher Scientific	Cat#SH30080.03
TD1 transposase and 2x transposase buffer	Illumina	Cat#FC-121-1030
Spermine	Sigma-Aldrich	Cat#S3256
Spermidine	Sigma-Aldrich	Cat#S2626
6AA	Sigma-Aldrich	Cat#A7824
Sucrose	Sigma-Aldrich	Cat#S7903
**Critical commercial assays**		
Foxp3/Transcription Factor Staining Buffer Set	ThermoFisher Scientific	Cat#00-5523-00
Ready-Set-Go IL-5 ELISA kit	ThermoFisher Scientific	Cat#88-7056-77
Ready-Set-Go IL-13 ELISA kit	ThermoFisher Scientific	Cat#88-7439-88

Table S2. **Key resources (*Continued*)**

Reagent or resource	Source	Identifier
Ready-Set-Go IL-17A ELISA kit	ThermoFisher Scientific	Cat#88-7176-76
Ready-Set-Go IL-22 ELISA kit	ThermoFisher Scientific	Cat#88-7522-88
Human IFN Gamma uncoated ELISA kit	ThermoFisher Scientific	Cat#88-7316-88
NucleoSpin RNA XS kit	Macherey-Nagel	Cat#740902.250
High-Capacity cDNA Reverse Transcription Kit	ThermoFisher Scientific	Cat#4368813
Clariom S pico Human HT 24-array plate	ThermoFisher Scientific	Cat#902963
QIAzol Lysis Reagent	Qiagen	Cat#79306
RNeasy kit	Qiagen	Cat#73404
GeneTitan™ Hybridization, Wash, and Stain Kit	Thermo Fisher	Cat#901622
iQ™SYBR®Green supermix	BioRad	Cat#64084532
Min–elute PCR purification kit	Qiagen	Cat#28004
**Deposited data**		
Micro-array data	This paper/MAD Dutch Genomics Service & Support Provider	GSE123817
ATAC-seq data	This paper	GSE124054
**Experimental models: Cell lines**		
Mouse: OP9 stromal cell line	Nakano, T. Osaka, Japan	N/A
Mouse: OP9-DL1 stromal cell line	Dontje et al, 2006	N/A
**Experimental models: Organisms/strains**		
N/A		
**Oligonucleotides**		
Primer: GATA3 forward: ACCACAACCACACTCTGGAGGA	This paper	N/A
Primer: GATA3 reverse: TCGGTTTCTGGTCTGGATGCCT	This paper	N/A
Primer: RORC forward: AATCTGGAGCTGGCCTTTCA	This paper	N/A
Primer: RORC reverse: CTGGAAGATCTGCAGCCTTT	This paper	N/A
Primer: TBX21 forward: ATTGCCGTGACTGCCTACCAGA	This paper	N/A
Primer: TBX21 reverse: GGAATTGACAGTTGGGTCCAGG	This paper	N/A
Primer: EOMES forward: AAATGGGTGACCTGTGGCAAA GC	This paper	N/A
Primer: EOMES reverse: CTCCTGTCTCATCCAGTGGGAA	This paper	N/A
Primer: GZMB forward: CGACAGTACCATTGAGTTGTGCG	This paper	N/A
Primer: GZMB reverse: TTCGTCCATAGGAGACAATGCCC	This paper	N/A
Primer: PRF1 forward: ACTCACAGGCAGCCAACTTTGC	This paper	N/A
Primer: PRF1 reverse: CTCTTGAAGTCAGGGTGCAGCG	This paper	N/A
Primer: GAPDH forward: GTCTCCTCTGACTTCAACAGCG	This paper	N/A
Primer: GAPDH reverse: ACCACCCTGTTGCTGTAGCCAA	This paper	N/A
**Recombinant DNA**		
N/A		
**Software and algorithms**		
GraphPad Prism 7.0	GraphPad	www.graphpad.com
FlowJo V10	FlowJo	www.flowjo.com
RStudio	RStudio	www.rstudio.com
Cytosplore +HSNE	Cytosplore	www.cytosplore.org
BioRad CFX Manager	BioRad	www.biorad.com
**Other**		

Table S2. **Key resources (*Continued*)**

Reagent or resource	Source	Identifier
Yssel's Medium	In house	N/A

Table S1 is provided online as an Excel file.
